# Identification of RTS,S/AS01 vaccine–induced humoral biomarkers predictive of protection against controlled human malaria infection

**DOI:** 10.1172/jci.insight.178801

**Published:** 2024-10-08

**Authors:** Rachel L. Spreng, Kelly E. Seaton, Lin Lin, Sir’Tauria Hilliard, Gillian Q. Horn, Milite Abraha, Aaron W. Deal, Kan Li, Alexander J. Carnacchi, Elizabeth Feeney, Siam Shabbir, Lu Zhang, Valerie Bekker, Sarah V. Mudrak, Sheetij Dutta, Laina D. Mercer, Scott Gregory, C. Richter King, Ulrike Wille-Reece, Erik Jongert, Neville K. Kisalu, Georgia D. Tomaras, S. Moses Dennison

**Affiliations:** 1 Duke Human Vaccine Institute,; 2 Center for Human Systems Immunology,; 3 Department of Surgery, and; 4 Department of Biostatistics and Bioinformatics, Duke University, Durham, North Carolina, USA.; 5 Walter Reed Army Institute of Research, Silver Spring, Maryland, USA.; 6 Center for Vaccine Innovation and Access, PATH, Seattle, Washington, USA.; 7 Center for Vaccine Innovation and Access, PATH, Washington, DC, USA.; 8 GSK, Rixensart, Belgium.; 9 Department of Integrative Immunobiology and; 10 Department of Molecular Genetics and Microbiology, Duke University, Durham, North Carolina, USA.

**Keywords:** Immunology, Vaccines, Malaria

## Abstract

**BACKGROUND:**

The mechanism(s) responsible for the efficacy of WHO-recommended malaria vaccine RTS,S/AS01 are not completely understood. We previously identified RTS,S vaccine–induced *Plasmodium falciparum* circumsporozoite protein–specific (PfCSP-specific) antibody measures associated with protection from controlled human malaria infection (CHMI). Here, we tested the protection-predicting capability of these measures in independent CHMI studies.

**METHODS:**

Vaccine-induced total serum antibody (immunoglobulins, Igs) and subclass antibody (IgG1 and IgG3) responses were measured by biolayer interferometry and the binding antibody multiplex assay, respectively. Immune responses were compared between protected and nonprotected vaccinees using univariate and multivariate logistic regression.

**RESULTS:**

Blinded prediction analysis showed that 5 antibody binding measures, including magnitude-avidity composite of serum Ig specific for PfCSP, major NANP repeats and N-terminal junction, and PfCSP- and NANP-specific IgG1 subclass magnitude, had good prediction accuracy (area under the receiver operating characteristic curves [ROC AUC] > 0.7) in at least 1 trial. Furthermore, univariate analysis showed a significant association between these antibody measures and protection (odds ratios 2.6–3.1). Multivariate modeling of combined data from 3 RTS,S CHMI trials identified the combination of IgG1 NANP binding magnitude plus serum NANP and N-junction Ig binding magnitude-avidity composite as the best predictor of protection (95% confidence interval for ROC AUC 0.693–0.834).

**CONCLUSION:**

These results reinforce our previous findings and provide a tool for predicting protection in future trials.

**TRIAL REGISTRATION:**

ClinicalTrials.gov NCT03162614, NCT03824236, NCT01366534, and NCT01857869.

**FUNDING:**

This study was supported by Bill & Melinda Gates Foundation’s Global Health-Discovery Collaboratory grants (INV-008612 and INV-043419) to GDT.

## Abstract 2

<span>Prediction of RTS,S vaccine induced protection against malaria from antibody measures using </span><span>logistic regression models trained on data from prior RTS,S trials</span><span>.</span>

## Introduction

RTS,S/AS01 (RTS,S) is the first World Health Organization–recommended (WHO-recommended) malaria vaccine for widespread use among children living in malaria-endemic settings (1, 2). This vaccine is administered with the AS01 adjuvant and has a truncated form of *Plasmodium* *falciparum* (*Pf*) circumsporozoite protein (PfCSP) containing the NANP major repeats and C-terminal region. PfCSP is localized to the surface of the sporozoites and is necessary for sporozoite development in the mosquito and invasion of hepatocytes, essential for initiating *Pf* infection (3–6). Phase III trials in Africa with RTS,S showed a reduction in malaria episodes by 53.9% (7) in children and 32.9% in infants over the course of 14 months (8). For participants who received a 4-dose regimen of RTS,S at months 0, 1, 2, and 20, vaccine efficacy (VE) decreased to 36.3% and 25.9% after 48 and 38 months from the first dose among children and infants, respectively (9). While numerous scientific breakthroughs, including the RTS,S vaccine, have lowered the morbidity and fatality rates associated with *Pf* malaria infection, progress in maintaining this decline has stalled (10) and new malaria cases have increased in some settings (1, 2). Thus, there is a need for new approaches to malaria interventions, including developing vaccines with improved efficacy and durability to minimize community transmission, with the ultimate goal of malaria eradication (1, 2, 11).

To improve VE and confer durable protection through rational vaccine design, it is critical to identify and validate correlates of protection (CoPs), i.e., immune biomarkers that exhibit statistically significant association with vaccine-mediated protection for a relevant clinical outcome such as infection or disease (12–14). Predictive analytics leveraging pre-identified CoPs from historical data is a powerful approach previously used to expedite vaccine development both across formulations, as during the selection of yearly influenza vaccine candidates (15), and across target populations, as during the bridging of VE across populations for dengue and COVID-19 vaccine candidates (16–18). The criteria used to benchmark vaccine performance or bridge VE across populations require reproducible CoPs to be identified in prior bridging trials, ensuring a reasonable likelihood of achieving a similar immune response and VE. Although a few mechanisms of RTS,S-mediated protection against *Pf* sporozoite challenge have been identified (19–21), currently there is no accepted antibody CoP for malaria vaccine candidates across different studies. Markers of protection identified in controlled human malaria infection (CHMI) and field (malaria-endemic regions) trials of RTS,S include total IgG against the major NANP repeats (22–25), IgG1 and IgG3 antibody subclasses targeting the PfCSP C-terminal region and NANP repeats (26), and avidity of antibodies for the PfCSP C-terminal region (22, 27, 28) and binding to Fcγ receptors (FcγRs) (19, 21). As such, integrated system serology profiling across CHMI and field trials will remain critical for validating CoP candidates.

Previously, we analyzed serum samples from 2 CHMI trials, ClinicalTrials.gov NCT01366534 (referred to as MAL068) (24) and NCT01857869 (referred to as MAL071) (25), to characterize RTS,S antibody–mediated protection. We studied antibody binding to key targets on PfCSP, including the NANP repeats and the N-terminal junction, even though it was not a part of RTS,S. Specifically, we investigated the specificity, magnitude, and avidity of total serum antibodies (include all isotypes, Ig) binding by biolayer interferometry (BLI) and binding of IgG subclass antibodies by employing a binding antibody multiplex assay (BAMA) (27, 28). Through univariate and multivariate modeling of the data from MAL068 and MAL071, we identified candidate antibody CoPs for RTS,S (28) associated with RTS,S vaccine–induced protection. In the present study, we hypothesized that these antibody CoP candidates would correlate with protection status in 2 recent CHMI studies involving RTS,S, i.e., MAL092 (ClinicalTrials.gov NCT03162614) and its follow up study MAL102 (ClinicalTrials.gov NCT03824236) (29, 30). Confirming these CoP candidates across CHMI trials can inform the design of next-generation malaria vaccines by providing precise measures for using as benchmarks to improve efficacy and durability of RTS,S-induced antibodies. Additionally, these candidate CoPs and other antibody measures analyzed herein can be used to support modifications of dose and schedule, or equivalence testing of product generated by different manufacturers.

Here, serum antibody binding was measured through biophysical assays, including BLI and BAMA. These measurements were then used to make blinded predictions of protection for each participant using the previously established univariate and bivariate modeling approaches (28). Univariate logistic regression using the MAL092 day of challenge (DoC) data was additionally performed to describe the association of antibody measurements with protection in this study. We then used penalized logistic regression with combined data from MAL092 (29, 30), MAL068 (24), and MAL071 (25) to identify a multivariate model that best predicted protection. Additionally, we analyzed differences in antibody responses associated with 2-dose or 3-dose regimens in MAL092 and assessed the durability of vaccine-induced humoral immune responses in the MAL102 trial. The results shown confirm a set of predictive biomarkers in CHMI trials and highlight a potential next step for testing these RTS,S vaccine–induced antibody CoP candidates in malaria-endemic field trials.

## Results

**Figure 1:**
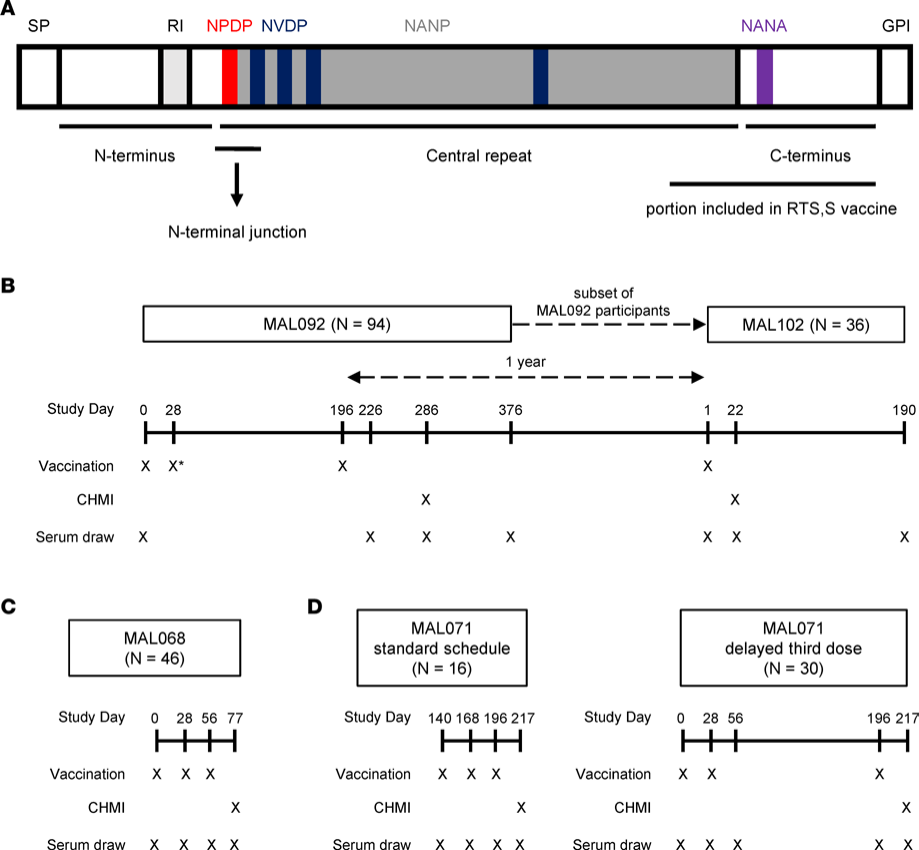
Schemas and study schedules. (**A**) Schematic of different domains in PfCSP. The major repeats of the NANP motif are shaded in gray and the interspersed minor repeats of the NVDP motif are shaded in blue. The location of the N-terminal junction region of the CSP-containing NPDP motif (shaded in red), which is referred to here as N Interface, is indicated. The portion of CSP included in RTS,S vaccine is indicated. Schedule of vaccination, CHMI, and serum draws tested for humoral immunity are shown for MAL092 and MAL102 (**B**), MAL068 (**C**), and MAL071 (**D**). *The MAL092 Adu1Fx group was not vaccinated on day 28.

The immunogenicity assessments and the efficacy of RTS,S in 2 recent CHMI clinical trials, MAL092 and the extension study MAL102, were reported previously (29, 30). In the present study, we assessed the magnitude, avidity, and specificity of vaccine-elicited antibody responses to PfCSP (Figure 1A) antigens in these 2 CHMI RTS,S clinical trials (study schemas in Table 1 and Figure 1B). As shown in Table 1, there were 5 treatment groups (29, 30). The AduFx group received an adult formulation of RTS,S and AS01_B_ adjuvant. The 2PedFx group received 2 times the volume of pediatric formulation of RTS,S and AS01_E_ adjuvant. The PedFx group received the pediatric formulation of RTS,S, and AS01_E_ adjuvant. These 3 groups received vaccination at 0, 1, and 7 months, with the third dose being fractional (1/5 of the full dose). The Adu2Fx group received the same formulation as AduFx, but the second and third doses were fractional (Table 1). Adu1Fx was the lone 2-dose regimen of the same formulation as AduFx vaccinated at months 0 and 7, with the last dose a fractional one (Table 1). The results of our assessment are shown in Supplemental Figures 1–5 and Supplemental Table 2 and 3; supplemental material available online with this article; https://doi.org/10.1172/jci.insight.178801DS1 Binding magnitude (response [nm]), dissociation rates (*k_d_* [s^–1^]), and magnitude-avidity composite calculated as the area under the dissociation curve in BLI assays (AUC_diss_ [nm × s]) for serum Igs at all study time points (measured by BLI) are shown in Supplemental Figures 1–3, respectively, with response rates and AUC_diss_ median and range summarized in Supplemental Table 2. The binding magnitude and avidity index (AI) for IgG1 and IgG3 subclasses for all study time points (measured by BAMA) are shown in Supplemental Figures 4 and 5, respectively. Response rates and antibody concentration expressed as monoclonal antibody (mAb) equivalence units (calculated relative to the PfCSP-specific mAb 334-hIgG1 or 334-hIgG3) based on BAMA data are summarized in Supplemental Table 3. We examined correlations among the measured immune responses and results (Supplemental Figure 6 and Supplemental Table 4) (28).

Overall, the highest antibody responses in serum were observed to the recombinant PfCSP, followed by responses to NANP6 (Supplemental Figures 1, 3, and 4). All antibody responses were readily boosted between prevaccination and the first postvaccination time point in MAL102 (Supplemental Figures 1, 3, and 4). Antibody avidity as measured by BLI (dissociation rates) and BAMA (AI) was maintained between MAL092 and MAL102 (Supplemental Figures 2 and 5).

We focused this analysis on antibody measurements that were significantly associated with protection in 2 prior independent CHMI RTS,S/AS01 trials, MAL068 and MAL071 (study schemas in Figure 1, C and D) (28). Here, we hypothesized that these prespecified antibody measurements (biomarkers) are associated with protection status in the current trials. These biomarkers included serum Ig magnitude-avidity composite (AUC_diss_) to full-length CSP, the central repeat region peptide NANP6, and the N Interface peptide corresponding to the N-terminal junction region of CSP using BLI (Figure 1A and Supplemental Table 1). The N-junctional region is not a part of the RTS,S vaccine, but was included in the analysis here for 2 reasons. Firstly, we previously observed cross-reactivity of both polyclonal antibodies (pAbs) and mAbs from RTS,S vaccinees with this peptide (27, 28, 31, 32), and secondly, we observed association of N-junctional region–specific serum antibodies magnitude with protection (27, 28). RTS,S-induced antibodies specific for the C-terminal region of PfCSP did not correlate with protection in our previous studies and, therefore, was not included for antibody measurements. AUC_diss_ is a composite measure of the magnitude of antigen-bound antibodies and their dissociation rate from the antigen. The subclass antibody biomarkers include magnitude of CSP-specific IgG1, NANP6-specific IgG1, and NANP6-specific IgG3. These are expressed in μg/mL equivalence units of an IgG subclass–matched standard mAb AB334 (334-hIgG1 and 334-hIgG3). Since IgG2 and IgG4 subclass measurements were associated with risk for malaria in RTS,S vaccinees (26) and did not correlate with protection in our previous study (28), they were not included in this analysis.

### Blinded prediction of protection in independent study cohorts.

**Figure 2:**
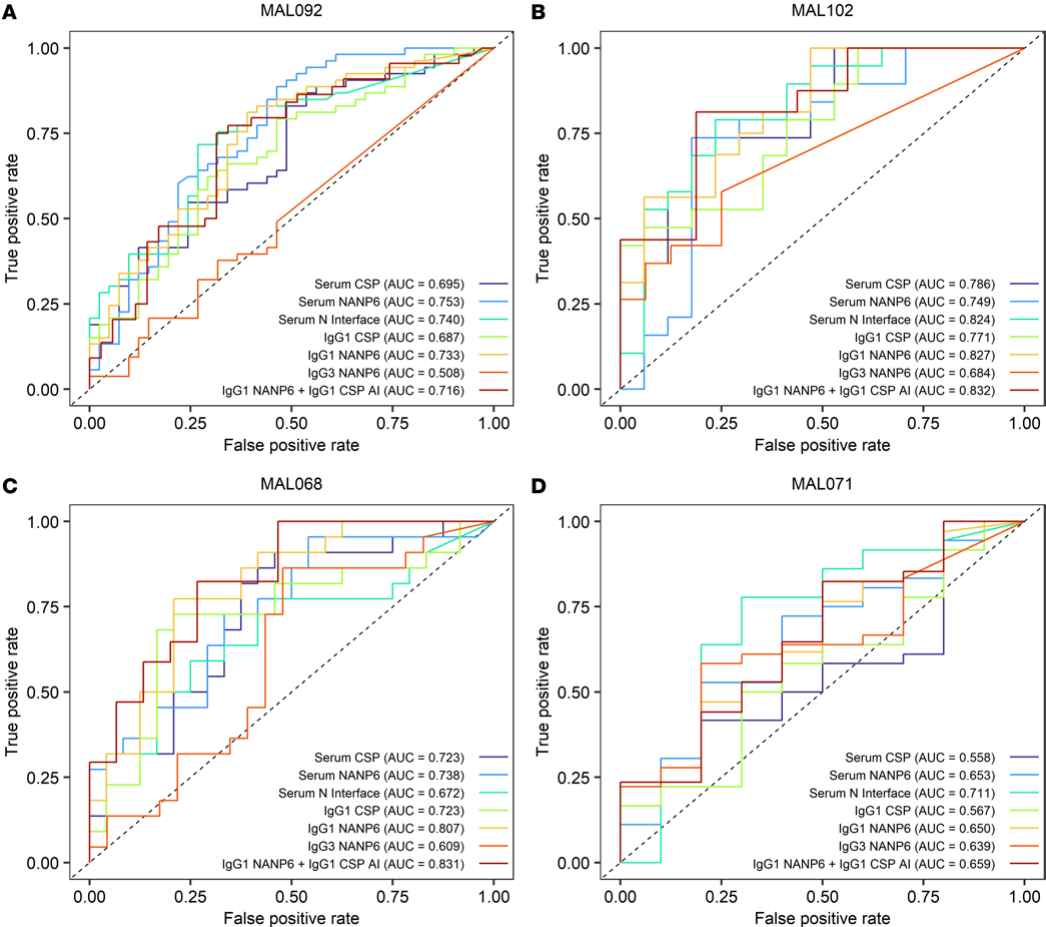
Predictive value of previously identified antibody biomarkers in an independent study population. ROC curves quantifying the ability of biomarkers individually and in combination to predict protection based on day of CHMI data are shown. Predictive models were trained using day of CHMI data from MAL068 and MAL071 combined and used to predict protection in MAL092 (**A**) and MAL102 (**B**), trained using day of CHMI data from MAL092 and MAL071 combined and used to predict protection in MAL068 (**C**), or trained using day of CHMI data from MAL092 and MAL068 combined and used to predict protection in MAL071 (**D**).

Previously identified univariate and multivariate logistic regression models (28) were used to predict protection from CHMI in MAL092 and MAL102 based on serum Ig magnitude-avidity composite and IgG subclass binding in samples collected on the DoC. These models were reanalyzed without regimen adjustment since the regimens in MAL092 and MAL102 were not all represented in the previous studies. ROC curves quantifying each biomarker’s ability to predict protection had AUC values of 0.508 to 0.753 in MAL092 (Figure 2A) and 0.684 to 0.832 in MAL102 (Figure 2B). For context, random prediction would result in a ROC curve with an AUC of 0.5, and a perfect CoP with no overlap in responses between protected and nonprotected vaccinees would result in a ROC curve with an AUC of 1. While there are not commonly accepted ROC AUC thresholds for goodness of prediction, we consider a ROC AUC of greater than 0.7 as good/acceptable and greater than 0.8 as excellent (33).

For both serum Ig and IgG1 binding in MAL092, ROC AUC values were larger for NANP6 (AUC of 0.753 for serum and 0.733 for IgG1) and N Interface (AUC of 0.740 for serum) models compared with CSP models (AUC of 0.695 for serum and 0.687 for IgG1). In both MAL092 and MAL102, IgG3 NANP6 was the least informative predictor with the lowest ROC AUC values, but was a better predictor in MAL102 (AUC 0.684) compared with MAL092 (AUC 0.508). The IgG1 NANP6 + IgG1 CSP AI multivariate model, identified as the best multivariate predictor using data from previous RTS,S studies (28), exhibited lower prediction probability than IgG1 NANP6 alone in MAL092 and only marginally better than IgG1 NANP6 alone in MAL102, suggesting that IgG1 CSP AI did not contribute new information to the prediction in MAL092 and MAL102. Three of the 6 univariate predictors (NANP6 and N Interface AUC_diss_ and IgG1 NANP6 334-hIgG1 equivalent concentration) had AUC values greater than 0.7 in both MAL092 and MAL102, suggesting that these biomarkers may be useful predictors of protection from CHMI.

As a sensitivity analysis of these biomarkers, reverse predictions were also tested, with logistic regression models either being trained on MAL071 and MAL092 and used to predict protection in MAL068 (Figure 2C) or trained on MAL068 and MAL092 used to predict protection in MAL071 (Figure 2D). Results were consistent with primary analyses, with 5 of the 6 univariate predictors having a ROC AUC of greater than 0.7 in at least one study, although no predictors had a ROC AUC of greater than 0.7 in both MAL068 and MAL071. This lower sensitivity of prediction across studies is likely due to the heterogeneity in the training set; MAL092 study participants received challenge at a much later time than either MAL068 or MAL071 vaccinees. In previous comprehensive analyses of MAL068 and MAL071, no univariate model had a cross-validation ROC AUC of greater than 0.75 (34). This highlights the complexity of identifying correlates of protection for RTS,S and, as suggested by Young et al. (34), may indicate differing mechanisms of protection dependent on the RTS,S regimen.

### Association of serum and IgG1 immune measures with protection from MAL092 CHMI.

**Figure 3:**
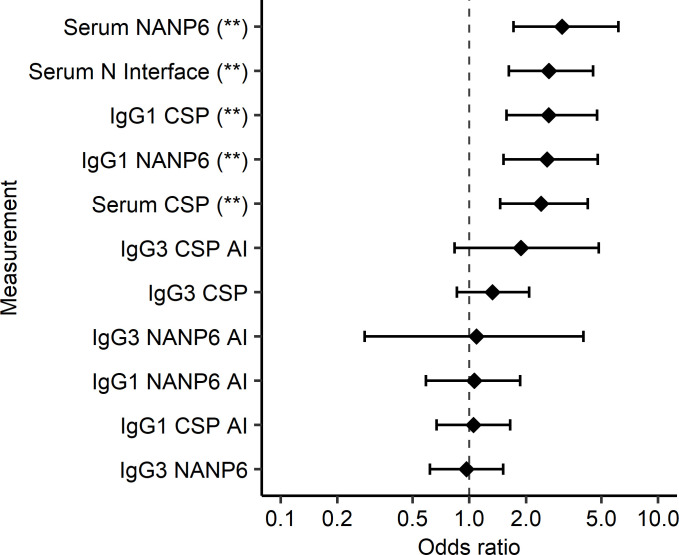
Serum Ig, IgG1 CSP, and NANP6 measurements associate with protection status in MAL092. Odds ratio estimates (circles) and 95% CIs (error bars) obtained from regimen-adjusted logistic regression models fit independently to each immune measurement (Protection ~ Regimen + immune measurement) based on MAL092 DoC data are shown. Asterisks represent statistically significant associations based on FDR-adjusted (Benjamini-Hochberg) *P* values: *P < 0.05, **P < 0.01.

**Figure 4:**
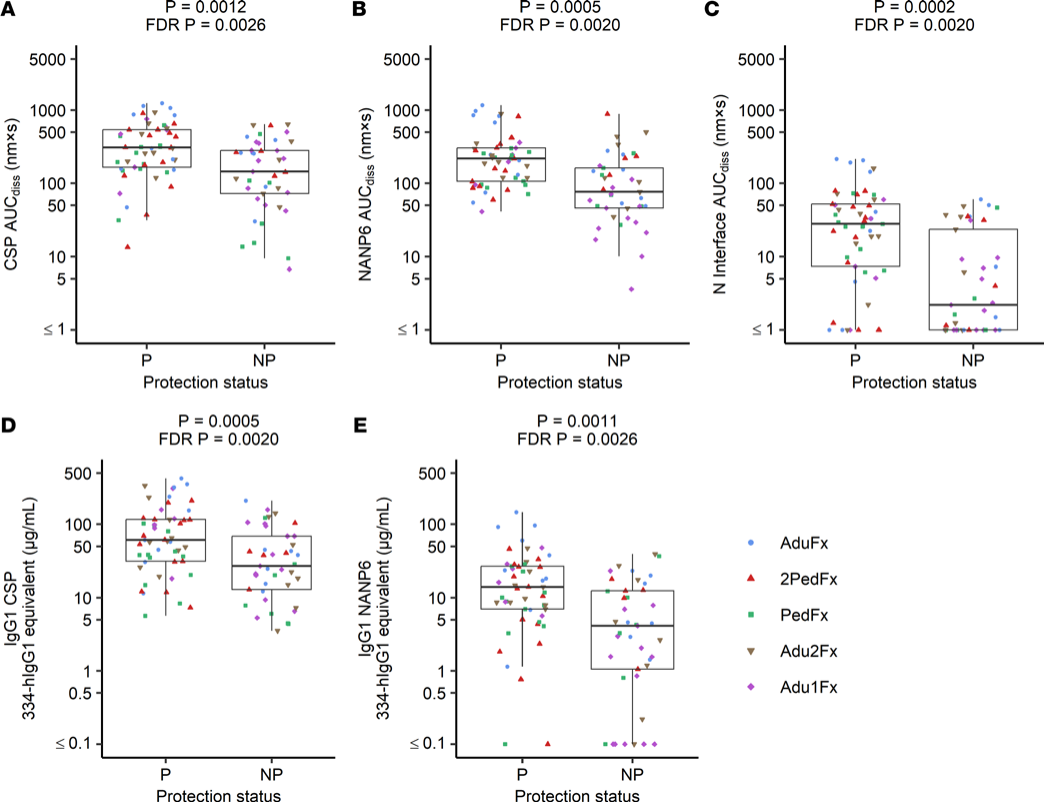
Serum Ig, IgG1 CSP, and NANP6 measurements associate with protection status in MAL092. Antibody measurements at DoC in the MAL092 study that have statistically significant associations with protection status based on regimen-adjusted logistic regression models fit independently to each immune measurement are shown. CSP (**A**), NANP6 (**B**), and N Interface (**C**) AUC_diss_, and IgG1 CSP (**D**) and NANP6 (**E**) 334-hIgG1 equivalent concentrations are compared for protected and nonprotected vaccinees (labeled as P and NP, respectively). Raw and FDR-corrected (Benjamini-Hochberg) *P* values shown from regimen-adjusted logistic regression models fit independently to each immune measurement. *n* = 94 (53 protected and 41 nonprotected). The lower and upper hinges of the box-and-whisker plots correspond to the 25th and 75th percentiles, with a line at the median. The lower and upper whisker extends from the box hinges to the smallest and largest values, respectively, which are within 1.5 × IQR of the hinge (where the IQR is equal to the distance between the 25th and 75th percentiles).

In addition to using prior models to perform blinded prediction of protection from CHMI in MAL092 and MAL102, regimen-adjusted logistic regression models were fitted independently to each immune measurement based on MAL092 DoC data. MAL102 data were not included in these analyses, both due to the smaller sample size and since MAL102 participants underwent CHMI in MAL092, introducing complications in interpreting the results. Results presented here are from regression models that included regimen as a covariate to account for differences in VE among regimen groups. Unadjusted models, which will be more generalizable, were also run, as well as models adjusted for age and sex in addition to regimen. Demographic variables did not contribute significantly to any models (*P* > 0.05) and the significance of associations between immune measures and protection from CHMI were unchanged in unadjusted models or in demographic-adjusted models (Supplemental Table 5). Serum Ig CSP, NANP6, and N Interface AUC_diss_ and IgG1 CSP and NANP6 334-hIgG1 equivalent concentration were each significantly associated with protection based on logistic regression, with FDR-adjusted *P* values of less than 0.005 (Figures 3 and 4, and Table 2) serving as additional evidence in support of these previously identified biomarkers (28). The Adu1Fx regimen, in which participants received only 2 doses (Table 1), indicator variable (1 = Adu1Fx vaccinee, 0 = non-Adu1Fx vaccinee) contributed significantly (*P* < 0.05) to 8 different single-biomarker models, including IgG1 CSP and NANP6 334-hIgG1 equivalent concentration, IgG3 CSP and NANP6 334-hIgG3 equivalent concentration, IgG1 and IgG3 CSP AI, and serum CSP and NANP6 AUC_diss_ models. In other words, none of these individual biomarkers are perfect predictors of protection and the addition of the Adu1Fx group assignment improves the prediction. This is consistent with the lower VE observed in the 2-dose Adu1Fx group compared with other 3-dose regimen groups (29), and also suggests that the lower efficacy in the Adu1Fx group cannot be fully explained by any of these individual immune biomarkers.

We also used the pAb avidity resolution tool (PAART) (35) to further understand the heterogeneity in avidity of RTS,S-induced pAbs between protected and nonprotected individuals based on the BLI binding kinetics data on MAL092 DoC. Due to the exclusion of all data below lower limit of quantitation (LLOQ) during PAART analysis, among CSP, NANP6, and N Interface, only binding to CSP was compared since the proportion of excluded data was lowest. Two dissociation rate components were identified for all vaccinees with CSP response greater than the LLOQ: a higher avidity (slower dissociation rate) component with a dissociation rate on the order of 10^–4^ s^–1^ and a lower avidity component with a dissociation rate on the order of 10^–2^ s^–1^. The median antigen occupancy by the higher avidity pAbs trended higher in protected vaccinees compared with nonprotected (96.7% vs. 95.8%, *P* = 0.073; Supplemental Figure 7), suggesting that high-avidity CSP-binding pAbs in serum may be associated with protection and could be explored in future trials with sufficiently large cohorts providing higher statistical power to assess this finding.

### Combinations of immune measures associated with protection in trial-combined data.

**Figure 5:**
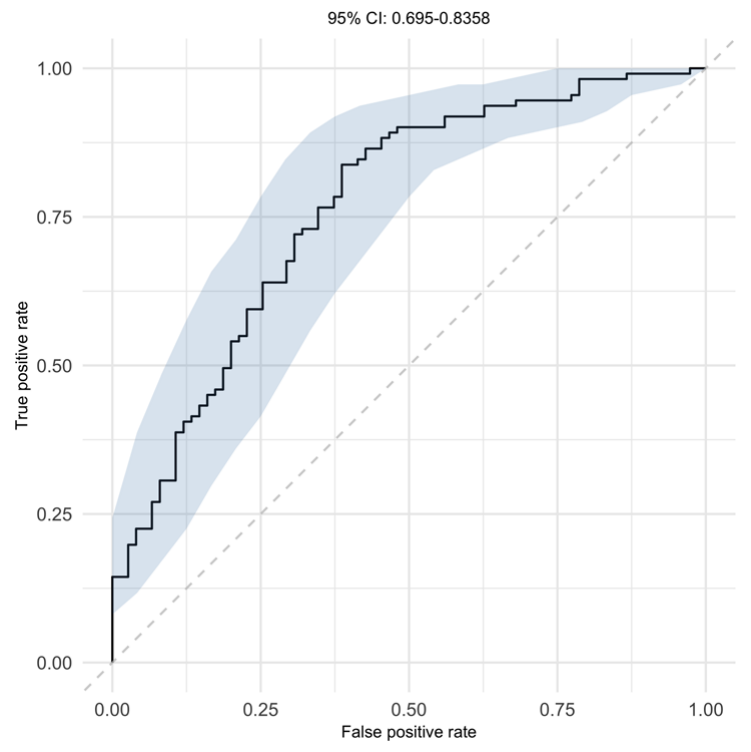
Serum NANP6 Ig, serum N Interface Ig, and IgG1 NANP6 measurements combined associate with protection status. Mean ROC curve (solid line) and 95% CI (shaded area) are plotted for best predictive model identified by 10-fold cross-validated logistic regression with LASSO penalty based on DoC data from MAL068, MAL071, and MAL092 combined.

To further explore combinations of immune measures, which mimics the situation in vivo and may provide improved prediction of protection from CHMI, logistic regression with least absolute shrinkage and selection operator (LASSO) penalty was performed using data from MAL092 combined with MAL068 and MAL071. Like in the univariate analysis, MAL102 data were not included in these analyses to avoid introducing the potential confounder of infection-induced immunity and complicating the interpretation of the results. In addition, the inclusion of repeated measures from participants in both MAL092 and MAL102 may introduce bias. The best model contained 3 variables, IgG1 NANP6 334-hIgG1 equivalent concentration, NANP6 and N Interface AUC_diss_, and had a 95% confidence interval (95% CI) of ROC AUC 0.693 to 0.834 based on 10-fold cross-validation (Figure 5). These results suggest that, for data combining multiple trials, a combination of immune biomarkers may be more predictive of protection than a single biomarker. While IgG1 PfCSP 334-hIgG1 equivalent concentration and PfCSP AUC_diss_ were significantly associated with protection in univariate models, they were not selected in the best multivariate model. This suggests that associations of IgG1 PfCSP 334-hIgG1 equivalent concentration and PfCSP AUC_diss_ with protection were not significant after accounting for the 3 variables included in the best model and that the PfCSP binding predominantly comprised the NANP binding. This is further supported by the observation that IgG1 PfCSP 334-hIgG1 equivalent concentration and PfCSP AUC_diss_ were highly correlated with the 3 variables included in the best multivariate model (Spearman’s *r* = 0.69 to 0.86, Supplemental Figure 6 and Supplemental Table 4). This is also consistent with the finding that univariate NANP6 and N Interface models had larger ROC AUC values (improved predictive power) compared with PfCSP models in MAL092 (Figure 2A).

### Regimen differences at DoC.

Having verified that 5 out of 6 of the previously identified biomarkers (28) indeed were also associated with RTS,S/AS01-induced protection in MAL092 (Figures 3 and 4, and Table 2), we next compared the biomarkers between regimens at DoC to identify key differences. On the DoC in MAL092 (90 days after final immunization), there were no significant differences in CSP AUC_diss_ or IgG3 CSP between regimens (*P* > 0.05, Supplemental Figure 8A and Supplemental Figure 9C, respectively). Vaccinees from the Adu1Fx arm (the only 2-dose regimen) had significantly lower NANP6 AUC_diss_ on the MAL092 DoC compared with all other regimens (*P* = 0.001 to 0.028, Supplemental Figure 8B), significantly lower N Interface AUC_diss_ and IgG3 NANP6 compared with the Adu2Fx arm (*P* = 0.045, Supplemental Figure 8C; *P* = 0.049, Supplemental Figure 9D), and significantly lower IgG1 NANP6 compared with the AduFx arm (*P* = 0.016, Supplemental Figure 9B). This indicates higher humoral immune responses induced by 3 doses of RTS,S compared with 2 doses, consistent with the primary immunogenicity analysis (29). We observed a few significant differences among the 3 dose regimens. Specifically, IgG1 CSP was significantly higher in the 2PedFx arm than in the PedFx arm (Supplemental Figure 9A). Both these arms received RTS,S adjuvanted with AS01_E_, but 2PedFx vaccines received twice the dose of RTS,S/AS01_E_ compared with PedFx vaccinees. There were no significant differences between the AduFx and 2PedFx arms, which received the same dose of RTS,S and AS01, but in half of the volume for AduFx compared with 2PedFx. Finally, there were also no significant differences between the AduFx and Adu2Fx arms, with the AduFx arm receiving only 1 fractional dose on day 196 (third immunization) and the Adu2Fx arm receiving fractional doses at both day 28 (second immunization) and day 196. Overall, these results are consistent with the primary immunogenicity data (29).

### Durability of RTS,S-induced antibody responses.

**Figure 6:**
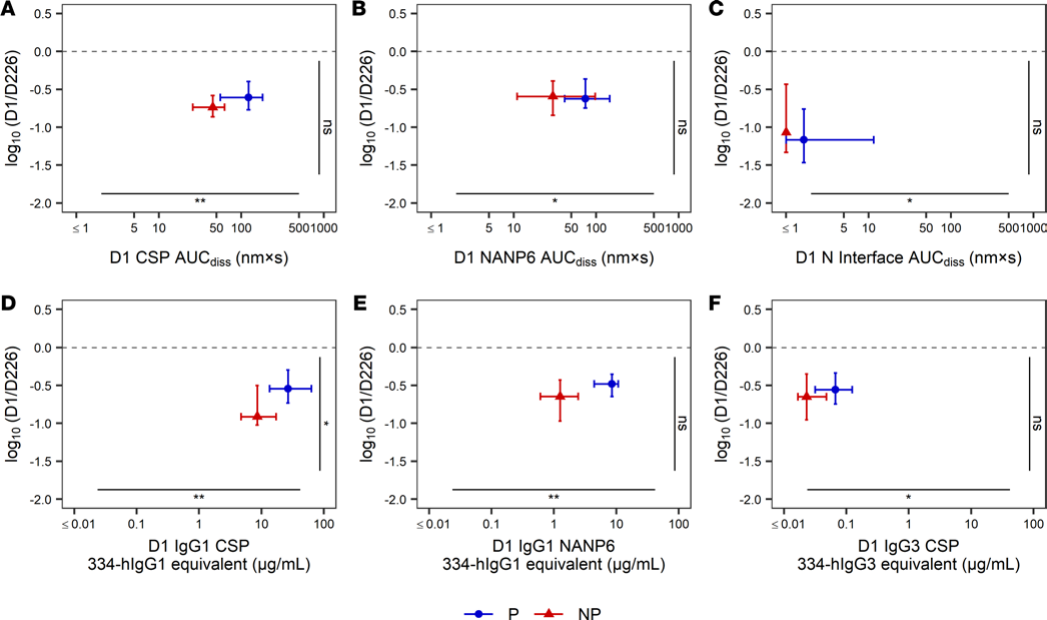
One-year durability of RTS,S/AS01-induced antibody responses. Fold change in antibody responses from MAL092 day 226 (30 days after the final immunization) to MAL102 day 1 (day of boost or 1 year after the final MAL092 immunization) versus MAL102 day 1 antibody responses by MAL092 protection status. PfCSP-specific (**A**), NANP6-specific (**B**), and N Interface–specific (**C**) AUC_diss_, and IgG1 CSP (**D**), IgG1 NANP6 (**E**), and IgG3 CSP (**F**) 334-hIgG1/hIgG3 equivalent concentrations. For MAL102 participants who were protected (P) or nonprotected (NP) from MAL092 CHMI, individual data points represent the median and error bars represent 25th to 75th percentiles. Horizontal dotted line represents no change in antibody response from MAL-092 day 226 to MAL-102 day 1. Asterisks along the horizontal and vertical axes indicate significant *P* values from Mann-Whitney *U* tests: *P < 0.05, **P < 0.01. NS, not significant.

We also assessed the durability of vaccine-induced antibody responses after 6 months and 1 year following RTS,S vaccination, respectively. Adu1Fx, the group with the lowest VE and the only 2-dose MAL092 regimen arm, had the least durable antibody responses after 6 months, with the lowest magnitude responses on day 376 (180 days after final immunization or 90 days after CHMI) for all measures except IgG1 CSP 334-hIgG1 equivalent and the largest fold declines between days 226 (30 days after final immunization) and 376 for all immune measures except N Interface AUC_diss_ (Supplemental Figure 10), suggesting that 3 doses may be necessary to induce robust and durable antibody responses. However, within the 3-dose regimen arms, there was not one regimen that consistently induced the most durable responses across all immune measures. MAL102 vaccinees who were nonprotected from CHMI in MAL092 had less durable responses 1 year after the final MAL092 immunization, with larger median fold declines in immune responses from MAL092 day 226 (30 days after final immunization) to MAL102 (day of boost or 1 year after final MAL092 immunization), with the exception of N Interface AUC_diss_ (Figure 6). N Interface AUC_diss_ had the largest decline by MAL102 day 1, with median log_10_(fold changes) of –1.17 and –1.07 in vaccinees protected and nonprotected from MAL092 CHMI, respectively. For all other immune measures, the median log_10_(fold change) ranged from –0.91 to –0.59 in vaccinees who were nonprotected from MAL092 CHMI and –0.62 to –0.48 in vaccinees who were protected from the first challenge (MAL092 CHMI). IgG1 CSP was the only measure with a significantly larger log_10_(fold change) in vaccinees nonprotected from MAL092 CHMI (*P* = 0.021, Figure 6D). All immune measures were significantly higher at MAL102 day 1 among vaccinees who were protected from MAL092 CHMI compared with vaccinees nonprotected in MAL092, with medians being 1.6- to 6.8-fold higher in vaccinees who were protected from MAL092 CHMI. Together, these results suggest that 3 doses of RTS,S vaccine induced robust, durable, and protective immune responses in vaccinees (64% protection across all 3-dose arms vs. 30% for Adu1Fx, i.e., the only 2-dose arm, within the participants included in these analyses) and that vaccinees who were protected from MAL092 CHMI had higher responses even at 1 year after the final dose. However, further testing of 3-dose regimens varying in dose and timing of immunizations may be needed to identify the most protective and durable responses.

## Discussion

CoPs are useful in rational design of vaccines for improved efficacy or durability, to bridge vaccines across populations, or as an endpoint for vaccine licensure if the CoP is agreed upon by regulatory agencies. An absolute correlate is a specific level (threshold) of response highly correlated with protection, whereas a relative correlate is a level of response that is variably associated with protection (12, 14). Identifying absolute CoPs from *Pf* infection is a challenging task compounded by the complexity of epitopes and immunodominant regions expressed on the *Pf* sporozoite, pathogenicity, multiple arms and effector function of the immune system, and the history of prior infections or exposures. As such, while absolute CoPs allow understanding the absolute efficacy of a vaccine and are therefore ideal as a basis of licensure, regulators frequently accept immune biomarkers that sufficiently correlate with efficacy and allow comparisons between groups and vaccines.

Vaccine-challenge CHMI studies in humans have been the primary strategy employed to identify a biomarker for malaria vaccine candidates. Previously, we identified multiple antibody biomarkers associated with RTS,S-induced protection (candidate CoPs) from homologous *Pf* infection in a CHMI model, including IgG1 binding to CSP, IgG1 and IgG3 binding to the central NANP repeat, as well as serum Ig AUC_diss_ for CSP, central NANP repeat peptide, and N-junctional epitope peptide binding (27, 28). The N-junctional epitope is not included in the RTS,S vaccine, but is targeted in addition to NANP repeats by protective mAbs induced by RTS,S (31, 32) and attenuated *Pf* sporozoites (36). We also showed that the N-junctional epitope–specific antibody binding was associated with protection in both MAL071 and MAL068 trials (27, 28). The N-junctional epitope peptide cross-reactivity of RTS,S-induced NANP-specific antibodies potentially would facilitate higher multivalent binding of CSP. In addition, this cross-reactivity of NANP-specific antibodies could result in an impediment of proteolytic processing of CSP due to the N-junctional region’s proximity to the proteolytic site to prevent CSP C-terminal exposure and its interaction with heparan sulfate proteoglycans on hepatocytes to facilitate the sporozoite invasion of these cells (37, 38). Additional biomarkers associated with protection against CHMI following RTS,S vaccination have been identified, including natural killer cell activation, antibody-dependent cell phagocytosis (ADCP), and engagement of FcγRIIIa (19, 21). The magnitude of the NANP-targeting antibodies, including the IgG1 and IgG3 subclasses, was found to be associated with protection in trials in endemic regions (22, 23, 26).

In this study, we have reduced the number of immune measures by investigating the same endpoint target (*Pf* infection) and similar biological targets (antibody magnitude, subclass, and avidity for select *Pf* antigens). Both the current and previous (28) studies identifying these candidate CoPs analyzed clinical studies that were completed in a similar challenge setting and participant population (i.e., CHMI of malaria-naive adults). Overall, our predictive analysis supports previous findings from our group (28) and others (34), suggesting that the biomarkers tested herein may be useful in future vaccine development, either alone or in combination with other immune measures such as ELISA titers, ADCP, and FcγR binding (19, 21, 34).

Although NANP-specific IgG3 was associated with protection in our prior analysis of MAL068 and MAL071 clinical trials (28), it was the least predictive biomarker in MAL092 and MAL102 and was not individually associated with protection in the MAL092 trial. This may be due in part to the 3-month time span between the final immunization and DoC in MAL092, resulting in many IgG3 binding responses falling below the LLOQ at DoC. This was likely a contributing factor for the inferiority of the IgG3 NANP6 model, particularly compared with prior analyses. Additionally, the relatively short half-life of IgG3 indicates that it may provide improved protection immediately after boosting when levels are highest. Moreover, current results also indicate a role for avid serum Ig and IgG1 CSP-specific antibodies in protection from *Pf* infection. This confirms findings observed in multiple trials of the RTS,S malaria vaccine in malaria-naive populations undergoing CHMI with a homologous challenge. Factors such as prior exposure to *Pf* and naturally occurring genetic polymorphisms have been shown to affect immunological responses to vaccines and VE (39, 40). Thus, assessment of these candidate biomarkers in individuals from malaria-endemic regions is needed for comparisons with previous findings (22, 23, 26) and to confirm correlation with protection to further probe vaccine-induced humoral immunity.

Beyond providing additional evidence for individual candidate CoPs, this study combines data from MAL068, MAL071, and MAL092 to explore the predictive power of a combination of biomarkers for protecting against CHMI. Other examples of predicting VE across formulations or populations include vaccines against influenza (15), dengue (16) and COVID-19 (17, 18). Influenza represents a pathogen with well-established biomarker guidelines to approve annual vaccine formulations (15). Biomarkers utilized in influenza vaccine analyses can be measured in any given influenza vaccine trial and are, therefore, readily applicable. Similar to the broad applicability of biomarkers in well-established influenza vaccine bridging, *Pf*-specific biomarkers identified herein can be applied, preferably after confirmation in the target population (endemic regions). Recent work to bridge a dengue vaccine into a new population (16) provides additional precedent for bridging CoPs across malaria vaccine trials, populations, and age groups. Although dengue has a better VE benchmark (41), translating protection using immune CoPs from CHMI trials in malaria-naive individuals to endemic regions is a major goal for the field (39). Future work is required to evaluate these candidate CoPs, identified in malaria-naive adults, in endemic populations, and in different age groups, particularly infants and children.

We also monitored the durability of the predictive biomarkers that correlated with protection and found the lowest efficacious 2-dose regimen, Adu1Fx, exhibiting the least durability at 6 months after the final immunization in MAL092 than the other 3-dose regimens, indicating the inferior outcome for the 2-dose regimen (Supplemental Figure 10). Interestingly, at 1 year after the last immunization, the protected vaccinees of MAL092 had significantly higher values of the antibody biomarkers and also exhibited higher durability of 4 out of 6 antibody biomarkers: CSP AUC_diss_, IgG1 CSP, IgG1 NANP6, and IgG3 CSP (Figure 6) compared with those vaccinees who were not protected. This observation is encouraging and can be explored further to correlate with VE. It will also be useful in developing next-generation vaccines to induce durable protective antibody responses.

While our findings show consistent associations between antibody magnitude, subclass, and avidity and protection across CHMI trials, the sample size was limited, though sufficient to identify statistically significant humoral measures associated with protection. Also, the MAL092 and MAL102 trials entail the use of homologous challenge in malaria-naive participants. As exposure in endemic areas can include multiple strains, investigating these results in a heterologous setting is necessary to determine the applicability of these candidate CoPs to a broader range of challenges, including settings where malaria transmission occurs perennially. Future work to reinforce these results or apply these candidate CoPs to larger study populations is necessary. Assessing functional activities associated with these biomarkers, such as antibody neutralization, antibody-dependent cellular cytotoxicity, or phagocytosis, may more specifically identify the mechanisms conferring protection and further refine our understanding of malaria CoPs. A parallel comprehensive analysis of antibody biomarkers and antibody-mediated functional biomarkers, when completed, is expected to illumine the role of antibody function and the underlying mechanism.

In summary, the association of the AUC_diss_ measurements with protection strongly suggests that antibody quality plays a role in protection in addition to the magnitude of the antibody level. We confirmed 5 prespecified antibody measures that exhibited high prediction accuracy in cross-trial analysis of protection from infection in CHMI clinical trials. These measures included IgG1 binding to the PfCSP major repeat NANP and magnitude-avidity composite (i.e., AUC_diss_) of serum antibodies binding to NANP and PfCSP N-terminal junction (association with protection, odds ratios 2.6–3.1). Furthermore, based on combined data from 3 independent RTS,S CHMI trials, the multivariate model containing IgG1 NANP binding magnitude and AUC_diss_ for serum NANP and N-junctional Ig was identified as the best predictor of protection, indicating that, in malaria infection, multiple immune biomarkers may be more predictive than individual biomarkers. Consistent association of these biomarkers with protection across trials provides increased confidence in the evaluation of these biomarkers in field trials in malaria-endemic settings and provides a tool to guide the development of next-generation malaria vaccines with improved efficacy.

## Methods

### Sex as a biological variable

This study examined data from both male and female individuals. The primary analyses described in this study did not consider sex as a biological variable. However, sex was included as a biological variable in the analyses presented in Supplemental Table 5, and we found that sex did not significantly contribute to predictive models.

### Samples and reagents

#### Study samples.

Samples from participants in the MAL092 and MAL102 clinical trials were collected following written informed consent (study schemas in Table 1). The efficacy and immunological evaluations for both clinical trials were reported previously (29, 30). Out of 130 participants originally enrolled in the MAL092 study (29), 94 participants who had previously provided consent for future use of their samples for research were included in this analysis (AduFx, *N* = 17; 2PedFx, *N* = 20; PedFx, *N* = 19; Adu2Fx, *N* = 18; and Adu1Fx, *N* = 20). For the MAL102 (30) analysis, serum samples from 36 participants from MAL092 reenrolled in MAL102 and who consented to use their samples for subsequent investigations were used (AduFx, *n* = 7; 2PedFx, *n* = 6; PedFx, *n* = 8; Adu2Fx, *n* = 6; and Adu1Fx, *n* = 9). All samples were deidentified.

#### Reagents.

Assay reagents, including antigens, positive controls, standards, and detection antibodies for BAMA and BLI, as appropriate are listed in Supplemental Table 1. A recombinant PfCSP and a synthetic peptide containing 6 NANP repeats (EP070334) were used in BAMA assays for measuring IgG1 and IgG3 antibody responses. PfCSP, biotinylated peptides with 6 NANP repeats (NANP6), and N-terminal junctional region of PfCSP (N Interface) were used in BLI assays for measuring serum antibody responses. The following secondary detection antibodies were used in the BAMA assay: mouse anti–human IgG3, purified anti–human IgG1, goat anti–mouse IgG antibodies, and Human ads-PE. Additional details of these secondary reagents are listed in Supplemental Table 1.

### Experimental methods

#### BAMA.

Binding measurements of PfCSP antigens to PfCSP-specific pAbs in vaccinee sera were evaluated using BAMA, as previously described (28). Briefly, carboxylated fluorescent beads (Luminex Corporation) were covalently coupled to PfCSP antigens and subsequently incubated with vaccinee samples in assay diluent (PBS, 5% normal goat serum, 0.05% Tween 20, 1% Blotto milk). No-antigen beads were used in all assays to account for nonspecific binding. Prescreened human reference serum was used as a negative control in assays. Lastly, beads mixed with serum samples were washed in assay buffer and evaluated on a FLEXMAP 3D instrument (Luminex Corporation). Binding-magnitude results were expressed as background and negative bead–subtracted mean fluorescence intensity (MFI) and as subclass-specific human IgG1 (334-hIgG1) or IgG3 (334-hIgG3) mAb equivalents (μg/mL). Equivalence units in vaccinee samples were calculated using 5-PL logistic regression based on a monoclonal 334-hIgG1 or 334-hIgG3 standard curve run in the same assay. Positive responders were defined as samples with MFI greater than 100, MFI × dilution factor greater than the 95th percentile of all baselines within the study, and MFI × dilution factor greater than 3 times participant-specific baseline MFI × dilution factor. AI was calculated as the percentage retained binding in sodium citrate buffer at pH 4.0 (CIT) versus PBS buffer alone (i.e., AI = MFI [CIT]/MFI [PBS] × 100) by using a modified BAMA with the addition of a 15-minute incubation step (after sample binding) containing either PBS or CIT followed by washing. All assays were performed according to Good Clinical Laboratory Practice Guidelines (GCLP), with assay tracking via Levey-Jennings charts.

#### BLI avidity assay.

The RTS,S/AS01 vaccine–induced serum antibody binding responses and the dissociation rates of interaction with recombinant PfCSP, NANP6, and N Interface (junctional epitope) antigens (Supplemental Table 1) were carried out using an established method for studying malaria vaccine–induced antibodies (42), as used for previous RTS,S/AS01 CHMI studies (27, 28). BLI assays were carried out using Fortebio Octet HTX and Octet Red384 instruments and biosensors (Sartorius). Both data acquisition and analyses were performed with the United States Food and Drug Administration’s Title 21 Code of Federal Regulations Part 11 (FDA Title 21 CFR Part 11) compliant software versions (Data Acquisition 12.0 and Data Analysis HT 12.0 packages).

Vaccinee sera from both studies were tested in triplicate for antigen binding at 1:50 dilution. For PfCSP and NANP6 binding, serum samples were diluted in PBS pH 7.4 (Gibco/Thermo Fisher Scientific). For N Interface binding, serum samples were diluted in 1× kinetics buffer (Sartorius). Antigens NANP6 and negative control peptide C1 (Supplemental Table 1) were loaded onto streptavidin (SA) biosensors (threshold level set not to exceed Δλ = 1 nm). PfCSP and negative control ovalbumin were coupled to the amine-reactive (AR2G) biosensors (threshold level set not to exceed Δλ = 0.7 nm). For N Interface binding, both N Interface and C1 peptides were loaded onto SA biosensors with a threshold set not to exceed Δλ equal to 0.1 nm. The 1:50-diluted vaccinee sera binding to the parallel reference sensors immobilized with negative control antigens were subtracted to obtain antigen-specific binding time courses. Binding responses (Δλ averaged at the last 5 seconds of the association phase) and the dissociation rates of vaccinee sera binding were determined. Antigen-specific positivity limit (mean plus 3 times the standard deviation of reference human serum binding response) and LLOQ (antigen-specific binding response of a standard mAb at an empirically determined concentration above which dissociation rate can be measured reliably) were applied in quality controlling of data. This involved ensuring that the percentage coefficient of variation (%CV) in binding responses that are positive for a given antigen (response > antigen specific positivity limit) was less than 20 and the variation in dissociation rates was 2-fold or lower for sera with responses greater than the LLOQ. For correlation analyses and the summary values in all tables, positive responders with binding responses below the LLOQ were assigned a dissociation rate of 1 × 10^–2^ s^–1^. The AUC of the dissociation curve (AUC_diss_), a composite measure of binding magnitude and avidity, was estimated for the specific binding time course data by using the trapezoidal rule in the R package “caTools” (https://www.r-project.org/foundation/).

#### Analysis of pAb avidity heterogeneity.

We analyzed the dissociation phases of vaccinees’ sera binding to antigens with the pAbs avidity resolution tool (PAART) (35). This tool fits the dissociation-phase response curves using a sum of exponentials model to identify different dissociation rate components, reflecting different avidity of pAbs, as well as the responses associated with them. Data from at least 2 of the 3 replicates were analyzed for each sample, with statistical analysis performed on the mean of replicate PAART results. The antigen occupancy (fraction bound to antigen in %) by pAbs of different avidities was determined as a ratio of response associated with a given dissociation rate to the sum of responses from all dissociation rates resolved.

### Statistics

All statistical analyses were performed using R statistical software (R Foundation for Statistical Computing, Vienna, Austria).

#### Univariate analysis.

Protection status in MAL092 and MAL102 was predicted using binomial logistic regression models (Protection ~ Measurement) independently fit to each variable using combined data from MAL068 and MAL071 via the “glm” function from the R package “stats.” All data were used irrespective of the group differences to increase the statistical power. Models were not adjusted for regimen since the regimen groups in the prediction data sets (MAL092 and MAL102) were not all represented in the training data sets (MAL068 and MAL071). Models and predictions were based on the DoC data, log-transformed prior to analysis. Predictions of protection status in MAL092 and MAL102 were performed in a blinded manner without incorporating knowledge of true protection status. This blinded analysis was conducted as per a prespecified statistical analysis plan. The R package “ROCR” was used to generate ROC curves to assess the predictive performance of each biomarker.

To compare immune responses between protected and nonprotected vaccinees in MAL092, binomial logistic regression models were fit to each variable independently based on the DoC data, with an additional term in the model to adjust for regimen (Protection ~ Regimen + Measurement). While adjusting for regimen may not be desirable for a generalizable CoP, we chose to include this adjustment here to improve the model predictions. Sensitivity analyses, unadjusted models, and models adjusted for age and sex in addition to regimen were also fit to each variable, with results presented in Supplemental Table 5. Each immune measurement was log-transformed and standardized (scaled to have a mean of 0 and a standard deviation of 1) within the study prior to analysis so that odds ratio estimates for immune measurements would be comparable. The Benjamini-Hochberg procedure was used to control the false discovery rate (FDR), and differences were considered statistically significant if FDR-adjusted *P* values were less than 0.2 and *P* values were less than 0.05 before FDR adjustment.

Comparisons of immune responses between regimens, as well as comparison of antigen occupancy by high-avidity antibodies identified by PAART between protected and nonprotected vaccinees, were performed using the Mann-Whitney *U* test. All *P* values are 2-sided, and differences were considered statistically significant for *P* values less than 0.05. No adjustments were made for multiple testing due to the small sample sizes and exploratory nature of these comparisons.

For all box-and-whisker plots, including supplemental figures, the lower and upper hinges of the box correspond to the 25th and 75th percentiles, with a line at the median. The lower and upper whiskers extend from the box hinges to the smallest and largest values, respectively, which are within 1.5 times the interquartile range (IQR) of the hinge (where IQR is equal to the distance between the 25th and 75th percentiles).

#### Multivariate analysis.

Logistic regression with LASSO penalty (L1 regularization) was performed using the “glmnet” R package (43). Models were trained using data from DoC from MAL068, MAL071, and MAL092. Data was, log-transformed, standardized, and models were adjusted for dose regimens as described previously (28). Both IgG3 NANP6 binding magnitude and AI were excluded from the multivariate analysis due to the large percentage of missing or nonquantifiable data. For MAL092 data on the DoC, more than 50% of the IgG3 NANP6 binding responses were below the LLOQ and 80% of the IgG3 NANP6 AI values were indeterminant or arbitrarily set to 1 due to binding responses not meeting the assay positivity criteria. Missing values were imputed using the predictive mean matching method in the “mice” R package (44). A 10-fold cross-validation was used to select the tuning parameter for LASSO that controls the amount of shrinkage applied to the coefficients. The “pROC” R package was used to generate the ROC curve to assess the classification performance of the logistic regression. To quantify the uncertainty, a 95% CI for the ROC was computed using 2000 stratified bootstrap replicates using “pROC.”

### Study approval

The study protocols for the underlying clinical trials were approved by the Walter Reed Army Institute of Research (WRAIR) Institutional Review Board (IRB), and all participants provided written informed consent. The retrospective analysis presented in this study was performed with approval from the Duke Medicine Institutional Review Board for Clinical Investigations (Pro00104803).

### Data Availability

All data generated or analyzed during this study are included in this article (and its supplemental information files). The data set used to perform statistical analysis and modeling can be downloaded from http://doi.org/10.5281/zenodo.10144807 Supporting data, including values for all data points shown in graphs and mean values, are available in the supplemental Supporting Data Values file.

## Author contributions

RLS wrote the prespecified statistical analysis plan, performed statistical analysis, and wrote the manuscript. KES, SMD, and GDT provided study oversight, designed experiments, supervised staff, analyzed data, and wrote the manuscript. LL and LDM performed statistical analysis and edited the manuscript. AWD, KL, and LZ analyzed data and edited the manuscript. AJC, EF, STH, GQH, MA, and SS designed experiments, performed experiments, collected and analyzed data, and edited the manuscript. VB provided program management, reviewed data, and edited the manuscript. SVM, SG, and UWR provided program management and edited the manuscript. SD provided reagents and edited the manuscript. CRK edited the manuscript. UWR contributed to the experimental design. EJ designed experiments, contributed program oversight, and reviewed the manuscript for scientific input. NKK contributed program oversight, analyzed data, and edited the manuscript. RLS and KES are shared first authors. A rotation policy was used to assign authorship order among these co–first authors.

## Supplementary Material

Supplemental data

ICMJE disclosure forms

Supporting data values

## Figures and Tables

**Table 2:**
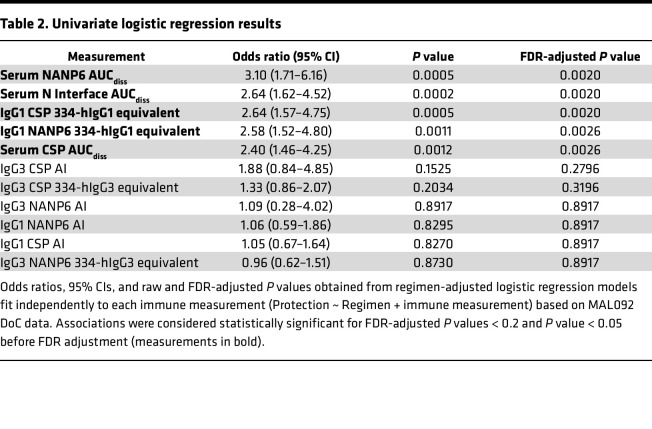
Univariate logistic regression results

**Table 1:**
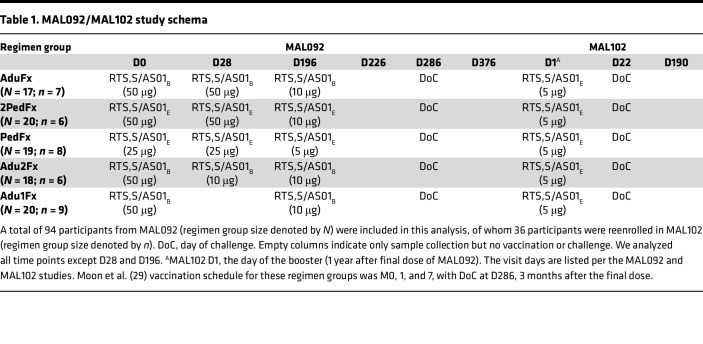
MAL092/MAL102 study schema

## Version 1 10/08/2024

Electronic publication
