# Supplementary material for: Identification of RTS,S/AS01 vaccine–induced humoral biomarkers predictive of protection against controlled human malaria infection
Source: JCI Insight. 2024 Oct 8;9(19):e178801. doi: 10.1172/jci.insight.178801 (PMC11466194; doi:10.1172/jci.insight.178801)
Supplement: Supplemental data [file jciinsight-9-178801-s198.pdf]

Supplemental Information

Supplemental Table 1. Reagents. List of reagents, including antigens, positive controls standards, and detection antibodies for BAMA and BLI.

Assay	Antigen	Epitope	Sequence	Source	Reference
BAMA, BLI	Recombinant CSP	N-terminal region, central repeat region and C-terminal region	MAHHHHHHPGMYGSSSNTRVLNELNYDNAGTNLYNELEMNYYGKQENWYS<br>LKKNSRSLGENDDGNNEDNEKLRKPKHKKLKQPADGNPDPNANPNVDPNAN<br>PNVDPNANPNVDPNANPNANPNANPNANPNANPNANPNANPNANPNANPN<br>ANPNANPNANPNANPNANPNANPNANPNKNNQGNGQGHNMPNDPNRNVD<br>ENANANSAVKNNNNEEPSDKHIKEYLNKIQNSLSTEWSPCSVTCGNGIQVRIK<br>PGSANKPKDELDYANDIEKKICKMEKCSSVFNVVNSGGRLEHHHHHH	Walter Reed Army Institute of Research, Silver Spring, MD	Schwenk, R., et al., PLoS One, 2014. 9(10): p. e111020
BAMA	EP070034	Central Repeat	NANPNANPNANPNANPNANPNANPC	Biomatik, Cambridge, ON, Canada	
BLI	NANP6	Central Repeat	biotin-Ahx-NANPNANPNANPNANPNANPNANP	CPC Scientific, Sunnyvale, CA	
BLI	N Interface	N-terminal junctional region	biotin-Ahx-KQPADGNPDPNANPN	CPC Scientific, Sunnyvale, CA	
BLI	C1	Negative control peptide	biotin-KKMQEDVISLWDQSLKPCVKLTPLCV	CPC Scientific, Sunnyvale, CA	
BLI	Ovalbumin	Negative control protein		Cytiva, Marlborough, MA	
**Assay**	**Monoclonal Antibodies**	**Clone Number**	**Catalog Number**	**Source**	
BAMA	Purified Anti-Human IgG1 Antibody	12G8G11	94796	BioLegend, San Diego, CA	
BAMA	Mouse Anti-Human IgG3	HP6047	53600	Life Technologies, Carlsbad, CA	
BAMA, BLI	AB334 IgG1	N/A	N/A	Duke Human Vaccine Institute, Protein Production Facility	Dennison et al J Immunol (2018) 201(4):1315-26, Regules et al J Infect Dis (2016) 214(5):762-71 and Williams et al Nat. Med. (2024) 30(1):117-129.
BAMA	AB334 IgG3	N/A	N/A	SriSai Pharmeceuticals	
**Assay**	**Polyclonal Antibodies**		**Catalog Number**	**Source**	
BAMA	Goat Anti-Mouse IgG, Human ads-PE		1030-09	BD Pharmingen, San Jose, CA	

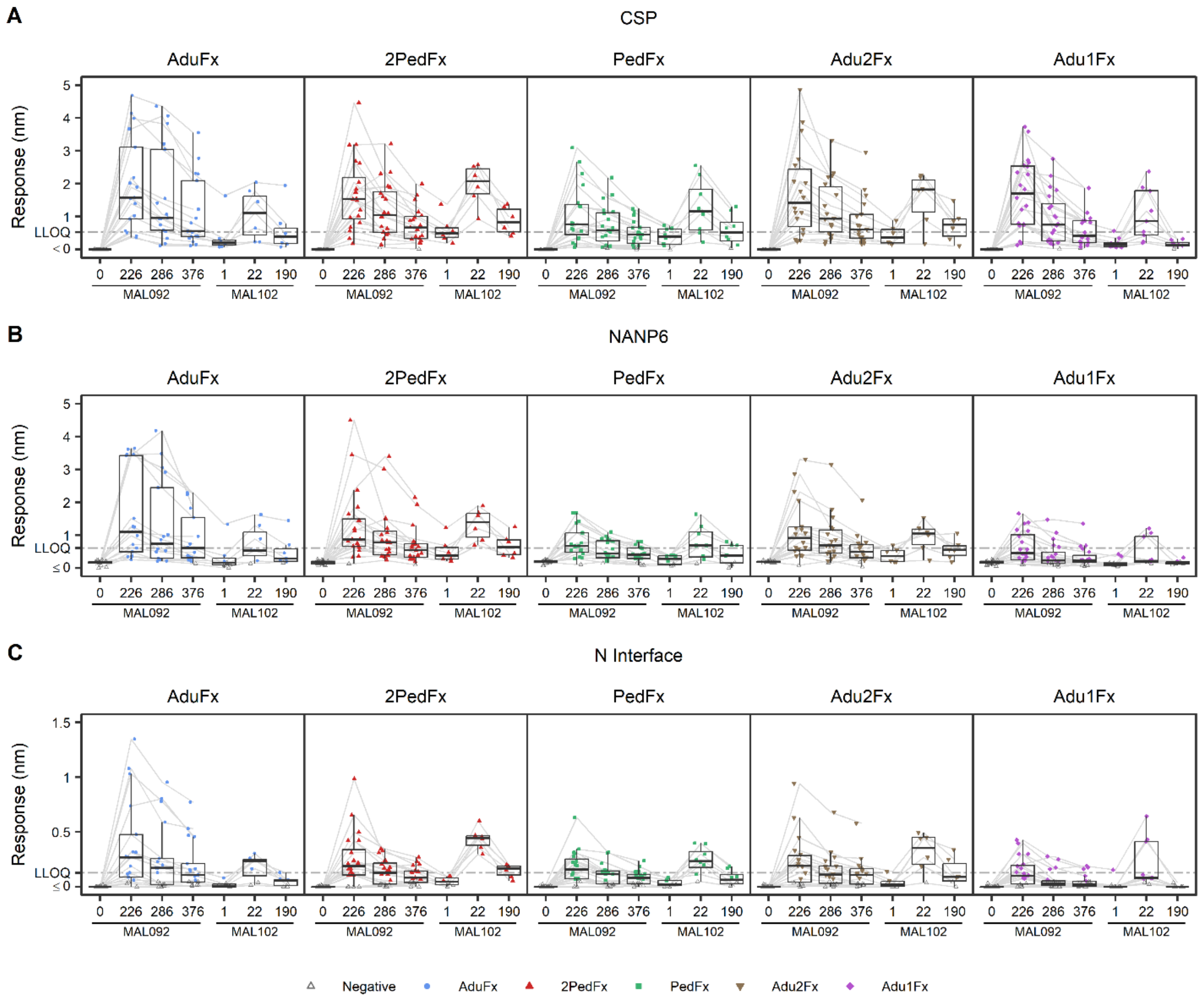

Supplemental Figure 1. Serum Ig binding responses induced by different RTS,S/AS01 regimens. Binding response of vaccinees' sera measured by BLI to CSP (**A**), NANP6 (**B**), and N Interface (**C**) antigens are shown for MAL092 days 0 (baseline), 226 (30 days post-final immunization), 286 (90 days post-final immunization or day of 1st CHMI), and 376 (180 days post-final immunization or 90 days post-1st CHMI) and MAL102 days 1 (day of boost or one year post-final MAL092 immunization), 22 (21 days post-boost or day of 2nd CHMI), and 190 (189 days post-boost or 168 days post-2nd CHMI). Note that panel C has a different y-axis scale compared to panels A-B to best show the change in response over time.

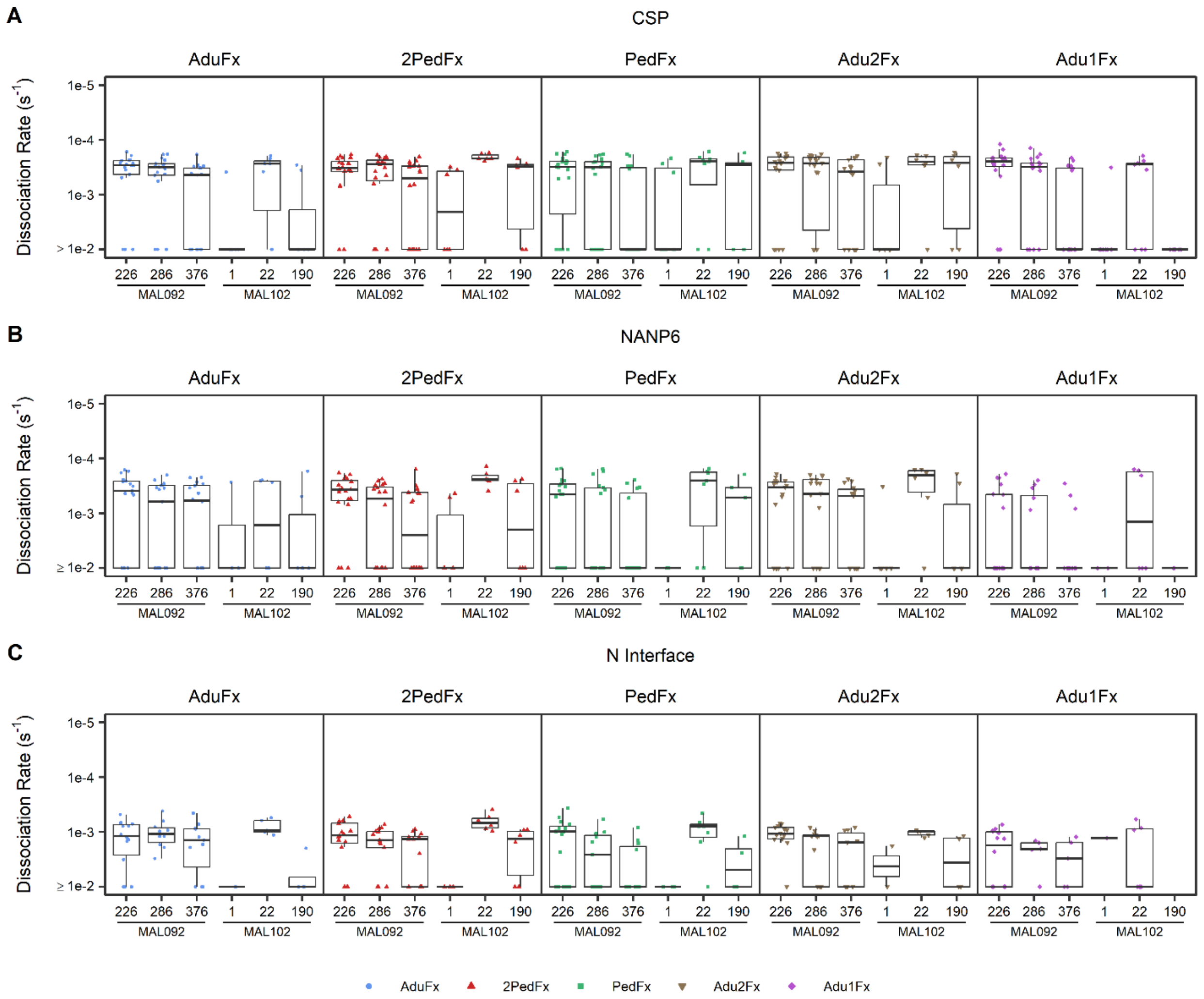

Supplemental Figure 2. Dissociation rates of serum Ig induced by different RTS,S/AS01 regimens. CSP-specific (**A**), NANP6-specific (**B**), and N Interface-specific (**C**) dissociation rates measured by BLI are plotted for MAL092 days 226, 286, 376 and MAL102 days 1, 22, and 190 as in S1 Fig. Dissociation rates are shown as ≥ 1e-2 for positive binding responses < LLOQ and are not shown for negative responses.

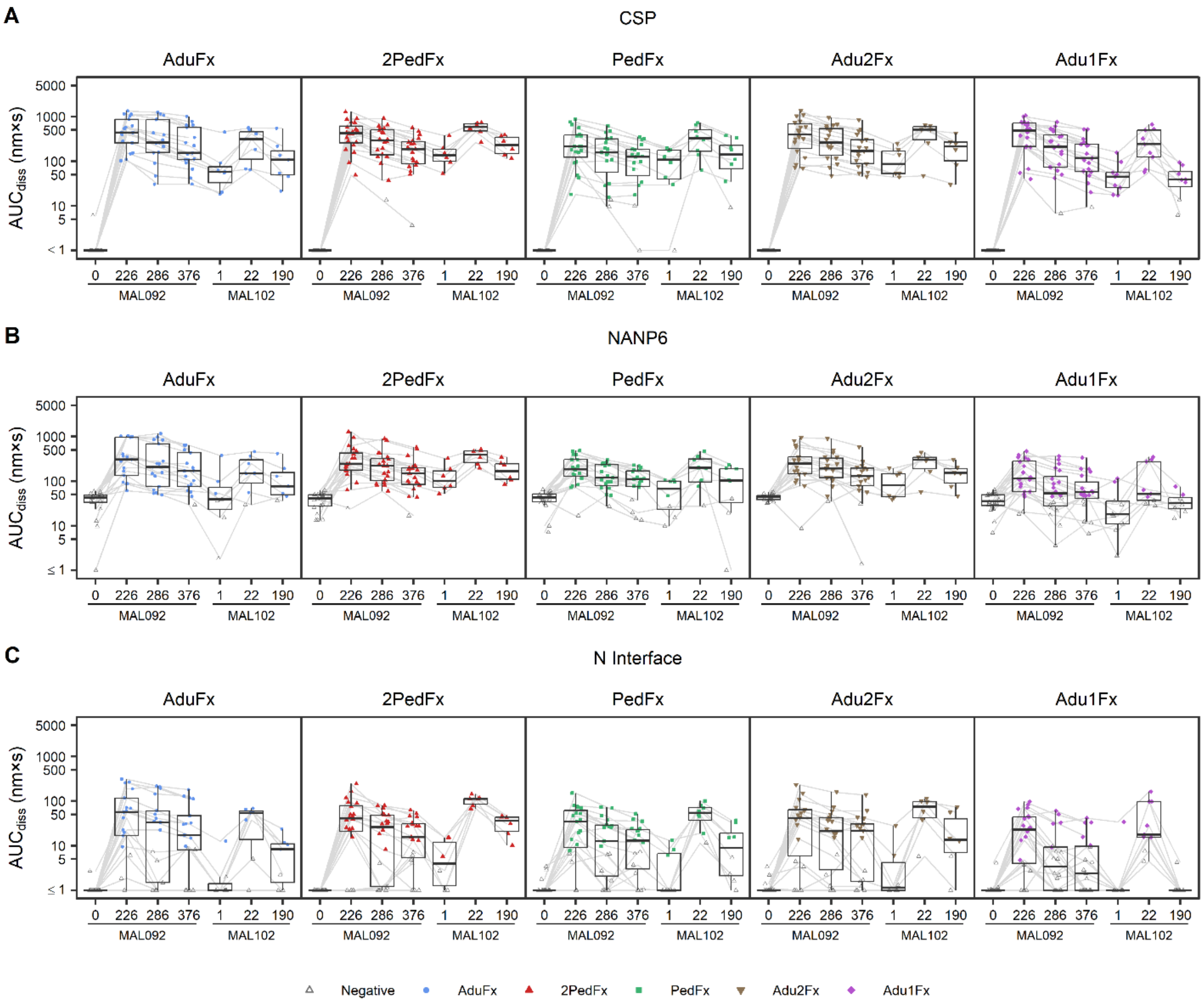

Supplemental Figure 3. Magnitude and avidity of serum Ig induced by different RTS,S/AS01 regimens. Plots show the CSP-specific (**A**), NANP6-specific (**B**), and N Interface-specific (**C**) AUC_{diss} measured by BLI at MAL092 days 0, 226, 286, 376, and MAL102 days 1, 22, and 190 as in S1 Fig.

Supplemental Table 2. Descriptive statistics for serum Ig binding magnitude and avidity in RTS,S/AS01 vaccinees from MAL092 and MAL102 studies. Response rates and median and range of AUC_{diss} (nm × s) measured by BLI are shown by study day and regimen. Response rates reflect the proportion of responses above the respective antigen-specific positivity limits as described in the Methods section.

Study Day	MAL092 Regimen	n	CSP		NANP6		N Interface	
			Response Rate	AUC_{diss} median (range)	Response Rate	AUC_{diss} median (range)	Response Rate	AUC_{diss} median (range)
MAL092								
0	AduFx	17	0.0%	0.0 (0.0, 6.1)	58.8%	42.7 (0.9, 58.2)	0.0%	0.0 (0.0, 2.7)
0	2PedFx	20	0.0%	0.0 (0.0, 0.0)	50.0%	40.5 (13.2, 62.2)	0.0%	0.0 (0.0, 1.0)
0	PedFx	19	0.0%	0.0 (0.0, 0.0)	57.9%	42.9 (7.1, 64.0)	0.0%	0.0 (0.0, 3.5)
0	Adu2Fx	18	0.0%	0.0 (0.0, 0.0)	66.7%	43.6 (32.0, 56.2)	0.0%	0.0 (0.0, 3.3)
0	Adu1Fx	20	0.0%	0.0 (0.0, 0.0)	50.0%	35.7 (7.0, 63.8)	0.0%	0.0 (0.0, 4.1)
226	AduFx	17	100.0%	439.4 (101.6, 1343.4)	100.0%	305.0 (61.4, 1045.1)	82.4%	56.3 (0.0, 310.0)
226	2PedFx	20	100.0%	426.9 (49.0, 1273.3)	95.0%	242.7 (24.2, 1277.4)	80.0%	41.1 (0.0, 244.5)
226	PedFx	19	100.0%	214.5 (18.1, 886.5)	94.7%	183.3 (16.7, 481.8)	78.9%	34.7 (0.0, 150.9)
226	Adu2Fx	18	100.0%	400.1 (69.8, 1368.8)	94.4%	249.6 (8.6, 944.9)	72.2%	41.6 (0.0, 232.5)
226	Adu1Fx	20	100.0%	486.8 (39.9, 1057.2)	90.0%	115.6 (18.3, 467.7)	70.0%	22.9 (0.0, 97.3)
286	AduFx	17	100.0%	261.5 (30.3, 1243.2)	100.0%	206.2 (49.1, 1165.6)	58.8%	33.1 (0.0, 215.4)
286	2PedFx	20	95.0%	292.5 (13.5, 907.9)	100.0%	219.8 (59.8, 886.6)	65.0%	26.4 (0.0, 79.3)
286	PedFx	19	89.5%	158.5 (9.5, 624.2)	94.7%	119.3 (27.1, 301.5)	57.9%	12.6 (0.0, 73.2)
286	Adu2Fx	18	100.0%	260.7 (47.4, 940.9)	94.4%	192.5 (34.9, 895.0)	66.7%	21.2 (0.0, 160.2)
286	Adu1Fx	20	95.0%	211.7 (6.8, 759.7)	60.0%	53.6 (3.6, 363.0)	25.0%	3.7 (0.0, 60.1)
376	AduFx	17	100.0%	152.6 (30.8, 1016.8)	94.1%	169.6 (29.5, 641.5)	64.7%	17.2 (0.1, 179.2)
376	2PedFx	19	94.7%	187.3 (3.7, 557.1)	94.7%	148.9 (16.9, 570.8)	63.2%	15.5 (0.0, 62.4)
376	PedFx	19	89.5%	125.5 (0.0, 352.4)	78.9%	110.0 (13.4, 226.9)	57.9%	13.0 (0.1, 52.1)
376	Adu2Fx	17	100.0%	169.7 (45.7, 841.1)	88.2%	130.3 (1.4, 579.2)	58.8%	21.6 (0.0, 138.5)
376	Adu1Fx	19	94.7%	117.2 (9.4, 522.5)	73.7%	56.6 (6.6, 336.5)	26.3%	2.4 (0.0, 41.6)
MAL102								
1	AduFx	7	100.0%	57.5 (18.3, 449.8)	42.9%	39.0 (1.8, 373.6)	14.3%	0.5 (0.0, 12.6)
1	2PedFx	6	100.0%	135.2 (53.1, 373.5)	100.0%	104.3 (52.3, 323.4)	50.0%	4.3 (0.0, 15.2)
1	PedFx	8	87.5%	109.7 (0.0, 198.4)	62.5%	72.2 (9.8, 105.6)	37.5%	0.7 (0.0, 13.3)
1	Adu2Fx	6	100.0%	96.2 (44.1, 251.3)	100.0%	92.2 (39.4, 195.2)	33.3%	0.7 (0.0, 28.1)
1	Adu1Fx	9	100.0%	44.9 (17.2, 157.4)	22.2%	18.1 (2.1, 114.6)	11.1%	0.0 (0.0, 34.1)
22	AduFx	7	100.0%	314.5 (64.7, 564.5)	85.7%	149.5 (29.7, 459.5)	71.4%	54.0 (0.3, 69.0)
22	2PedFx	6	100.0%	591.4 (265.9, 729.6)	100.0%	398.3 (197.0, 516.3)	100.0%	111.5 (67.3, 143.7)
22	PedFx	8	100.0%	327.9 (63.2, 720.4)	87.5%	197.2 (27.7, 467.6)	87.5%	54.4 (11.5, 100.8)
22	Adu2Fx	6	100.0%	512.4 (47.6, 638.6)	100.0%	299.3 (57.5, 438.1)	83.3%	76.7 (5.7, 115.2)
22	Adu1Fx	9	100.0%	242.7 (57.2, 667.5)	66.7%	51.9 (28.3, 349.1)	77.8%	17.8 (4.5, 162.5)
190	AduFx	7	100.0%	108.2 (21.4, 548.6)	85.7%	76.3 (36.2, 416.2)	57.1%	8.3 (0.0, 23.9)
190	2PedFx	6	100.0%	234.4 (114.7, 386.4)	100.0%	175.1 (84.8, 343.5)	100.0%	36.6 (10.2, 46.3)
190	PedFx	8	87.5%	145.4 (9.1, 374.8)	62.5%	102.8 (0.0, 233.9)	50.0%	10.2 (0.5, 37.1)
190	Adu2Fx	6	100.0%	215.4 (30.0, 422.9)	100.0%	152.7 (46.2, 304.6)	66.7%	13.6 (0.0, 73.9)
190	Adu1Fx	8	75.0%	38.8 (6.2, 93.9)	25.0%	32.1 (14.5, 75.6)	0.0%	0.0 (0.0, 4.2)

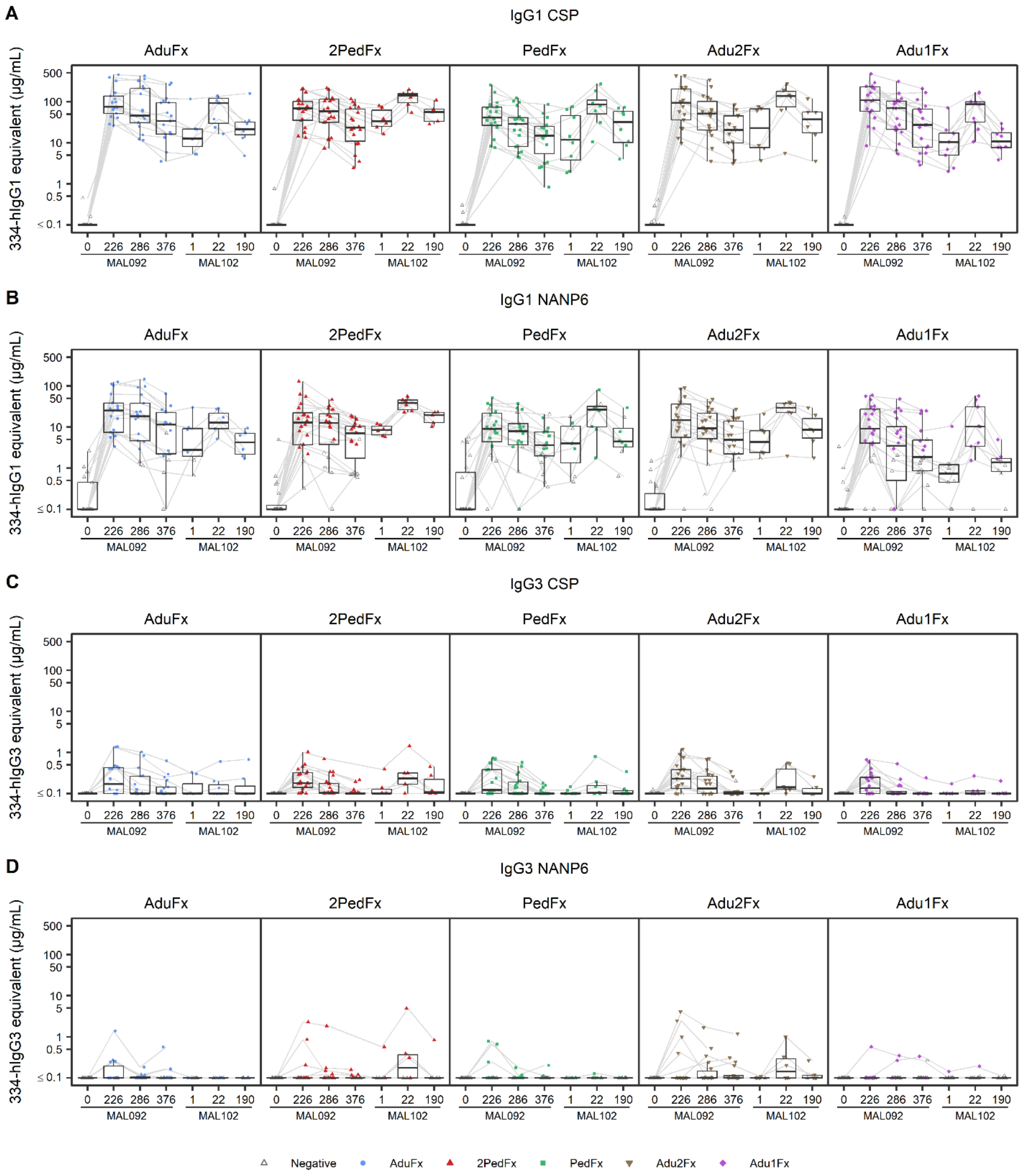

Supplemental Figure 4. Magnitude of RTS,S/AS01 induced antibody subclass binding in different regimens. Plots show the IgG1 CSP (**A**), IgG1 NANP6 (**B**), IgG3 CSP (**C**), and IgG3 NANP6 (**D**) 334-hIgG1/hIgG3 equivalent concentration measured by BAMA at MAL092 days 0, 226, 286, 376, and MAL102 days 1, 22, and 190 as in S1 Fig.

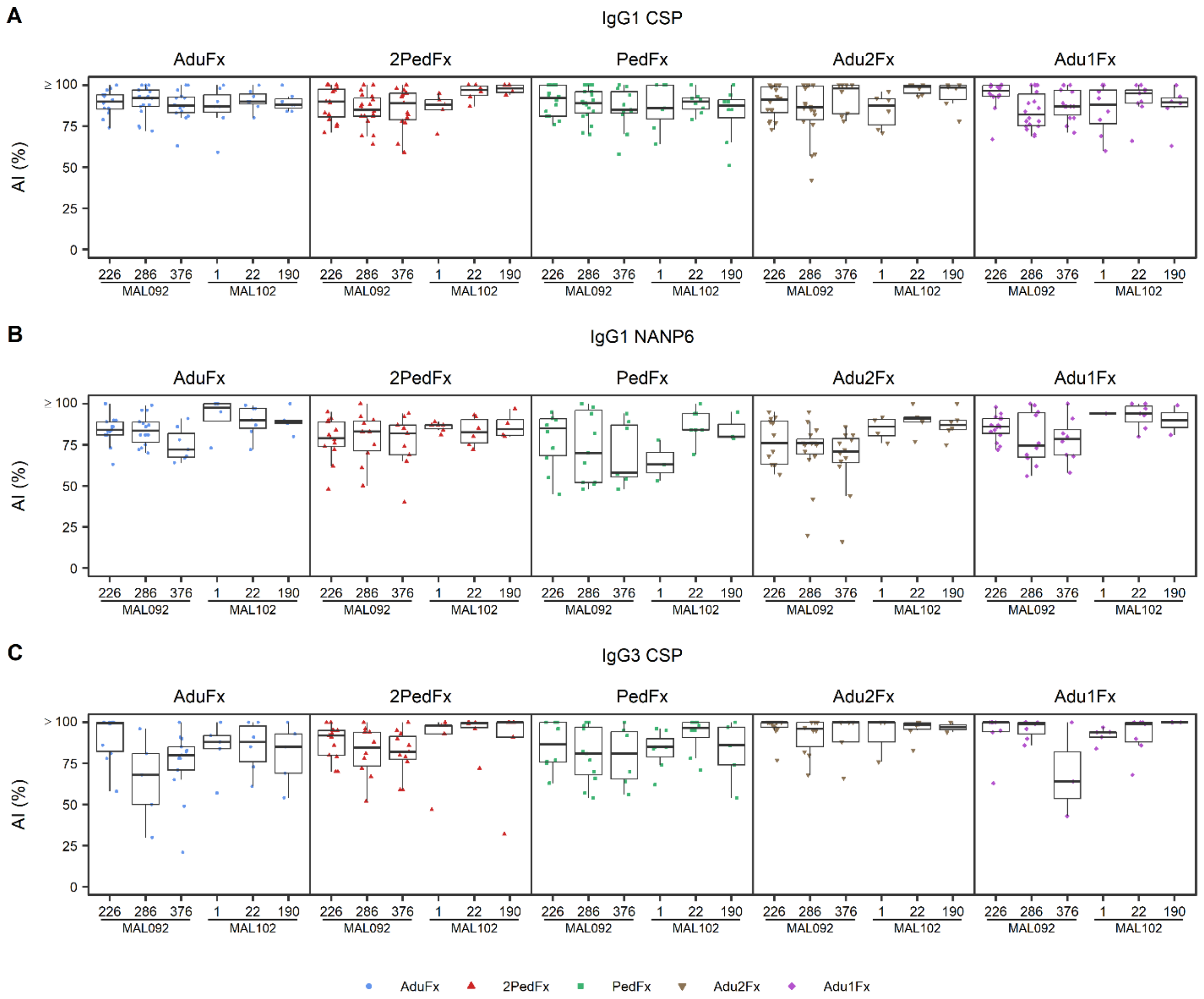

Supplemental Figure 5. Antibody subclass binding avidity in different RTS,S/AS01 regimens. Plots show the IgG1 CSP (**A**), IgG1 NANP6 (**B**), and IgG3 CSP (**C**) AI measured by BAMA at MAL092 days 226, 286, 376, and MAL102 days 1, 22, and 190 as in S1 Fig.

Supplemental Table 3. Descriptive statistics for antibody subclass binding magnitude in RTS,S/AS01 vaccinees from MAL092 and MAL102 studies. Response rates and median and range of 334-hIgG1/hIgG3 equivalent (µg/mL) measured by BAMA are shown by study day and regimen. Response rates reflect proportion of positive vaccine responders as defined in the Methods section.

Study Day	MAL092 Regimen	n	IgG1 CSP		IgG1 NANP6		IgG3 CSP		IgG3 NANP6	
			Response Rate	µg/mL median (range)	Response Rate	µg/mL median (range)	Response Rate	µg/mL median (range)	Response Rate	µg/mL median (range)
MAL092										
0	AduFx	17	0.0%	0.01 (0.01, 0.45)	0.0%	0.10 (0.10, 2.58)	0.0%	0.00 (0.00, 0.02)	0.0%	0.01 (0.01, 0.03)
0	2PedFx	20	0.0%	0.02 (0.01, 0.76)	0.0%	0.10 (0.10, 4.89)	0.0%	0.00 (0.00, 0.00)	0.0%	0.01 (0.01, 0.01)
0	PedFx	19	0.0%	0.01 (0.01, 0.30)	0.0%	0.10 (0.10, 5.50)	0.0%	0.00 (0.00, 0.06)	0.0%	0.01 (0.01, 0.01)
0	Adu2Fx	18	0.0%	0.01 (0.01, 0.40)	0.0%	0.10 (0.10, 1.49)	0.0%	0.00 (0.00, 0.12)	0.0%	0.01 (0.01, 0.03)
0	Adu1Fx	20	0.0%	0.01 (0.01, 0.15)	0.0%	0.10 (0.10, 3.33)	0.0%	0.00 (0.00, 0.08)	0.0%	0.01 (0.01, 0.05)
226	AduFx	17	100.0%	74.69 (25.95, 454.43)	100.0%	25.28 (3.37, 126.16)	100.0%	0.18 (0.06, 1.38)	87.5%	0.01 (0.01, 1.39)
226	2PedFx	20	100.0%	68.85 (13.14, 206.40)	85.0%	12.86 (0.32, 129.15)	100.0%	0.18 (0.01, 1.02)	68.4%	0.01 (0.01, 2.30)
226	PedFx	19	100.0%	41.03 (7.69, 255.56)	73.7%	9.00 (0.10, 51.67)	89.5%	0.12 (0.00, 0.73)	57.9%	0.01 (0.01, 0.79)
226	Adu2Fx	18	100.0%	93.51 (9.73, 425.28)	88.9%	14.97 (0.10, 90.56)	88.2%	0.23 (0.00, 1.21)	77.8%	0.01 (0.01, 4.09)
226	Adu1Fx	20	100.0%	108.30 (8.49, 476.31)	80.0%	9.16 (0.10, 59.68)	85.0%	0.14 (0.00, 0.67)	50.0%	0.01 (0.01, 0.57)
286	AduFx	17	100.0%	45.24 (11.51, 424.02)	88.2%	18.30 (1.15, 146.82)	76.5%	0.07 (0.00, 1.03)	58.8%	0.03 (0.01, 0.18)
286	2PedFx	20	100.0%	58.09 (7.33, 211.19)	70.0%	12.62 (0.10, 46.52)	75.0%	0.09 (0.00, 0.69)	45.0%	0.01 (0.01, 1.83)
286	PedFx	19	100.0%	28.66 (4.40, 122.52)	73.7%	7.93 (0.10, 37.02)	89.5%	0.10 (0.00, 0.57)	57.9%	0.03 (0.01, 0.18)
286	Adu2Fx	18	100.0%	51.29 (3.54, 337.26)	83.3%	9.22 (0.10, 47.61)	83.3%	0.13 (0.00, 0.70)	50.0%	0.03 (0.01, 1.68)
286	Adu1Fx	20	100.0%	69.27 (5.33, 310.51)	60.0%	3.55 (0.10, 48.31)	65.0%	0.05 (0.00, 0.53)	25.0%	0.01 (0.01, 0.35)
376	AduFx	17	100.0%	33.70 (3.53, 285.21)	70.6%	11.50 (0.10, 65.40)	94.1%	0.07 (0.01, 0.62)	52.9%	0.02 (0.01, 0.57)
376	2PedFx	19	100.0%	23.45 (2.44, 116.14)	68.4%	7.08 (0.58, 18.75)	89.5%	0.06 (0.00, 0.22)	52.6%	0.02 (0.01, 0.16)
376	PedFx	19	100.0%	14.83 (0.82, 86.56)	52.6%	3.62 (0.35, 20.64)	84.2%	0.04 (0.00, 0.18)	21.1%	0.01 (0.01, 0.20)
376	Adu2Fx	17	100.0%	20.59 (3.13, 84.35)	70.6%	4.89 (0.89, 27.03)	82.4%	0.06 (0.01, 0.35)	52.9%	0.04 (0.01, 1.20)
376	Adu1Fx	19	100.0%	27.15 (2.88, 207.81)	42.1%	1.85 (0.10, 25.26)	78.9%	0.03 (0.01, 0.24)	10.5%	0.01 (0.01, 0.34)
MAL102										
1	AduFx	7	100.0%	12.62 (5.25, 115.32)	71.4%	2.77 (0.65, 30.10)	85.7%	0.05 (0.01, 0.33)	42.9%	0.01 (0.01, 0.08)
1	2PedFx	6	100.0%	33.88 (16.21, 79.87)	100.0%	8.47 (5.74, 12.01)	100.0%	0.08 (0.03, 0.39)	33.3%	0.01 (0.01, 0.57)
1	PedFx	8	100.0%	12.04 (1.86, 75.35)	42.9%	3.99 (0.46, 17.88)	87.5%	0.03 (0.01, 0.15)	25.0%	0.01 (0.01, 0.06)
1	Adu2Fx	6	100.0%	34.40 (3.70, 77.77)	83.3%	5.01 (1.70, 21.01)	100.0%	0.05 (0.02, 0.12)	50.0%	0.04 (0.01, 0.11)
1	Adu1Fx	9	100.0%	10.44 (2.01, 70.78)	11.1%	0.73 (0.10, 10.39)	66.7%	0.02 (0.00, 0.17)	11.1%	0.01 (0.01, 0.14)
22	AduFx	7	100.0%	91.77 (16.43, 139.34)	100.0%	12.72 (5.11, 28.82)	100.0%	0.09 (0.06, 0.60)	28.6%	0.01 (0.01, 0.08)
22	2PedFx	6	100.0%	141.77 (53.87, 196.95)	100.0%	38.80 (23.05, 56.13)	100.0%	0.24 (0.09, 1.45)	66.7%	0.17 (0.01, 4.90)
22	PedFx	8	100.0%	85.10 (10.40, 269.62)	87.5%	26.75 (1.77, 81.36)	100.0%	0.10 (0.03, 0.79)	37.5%	0.01 (0.01, 0.13)
22	Adu2Fx	6	100.0%	137.98 (5.62, 275.03)	100.0%	30.18 (1.99, 39.99)	100.0%	0.14 (0.08, 0.57)	83.3%	0.13 (0.01, 1.00)
22	Adu1Fx	9	100.0%	86.39 (10.56, 169.03)	88.9%	10.26 (0.10, 57.24)	100.0%	0.08 (0.02, 0.27)	11.1%	0.01 (0.01, 0.19)
190	AduFx	7	100.0%	21.33 (4.78, 158.23)	100.0%	4.55 (1.70, 9.32)	100.0%	0.05 (0.02, 0.67)	28.6%	0.01 (0.01, 0.06)
190	2PedFx	6	100.0%	55.68 (28.13, 107.81)	100.0%	19.38 (10.28, 23.33)	100.0%	0.10 (0.04, 0.45)	33.3%	0.01 (0.01, 0.83)
190	PedFx	8	100.0%	34.52 (3.96, 72.07)	62.5%	4.43 (0.64, 29.83)	100.0%	0.04 (0.02, 0.34)	25.0%	0.02 (0.01, 0.05)
190	Adu2Fx	6	100.0%	39.90 (3.59, 117.90)	100.0%	8.60 (1.79, 29.60)	100.0%	0.08 (0.03, 0.26)	83.3%	0.08 (0.01, 0.27)
190	Adu1Fx	8	100.0%	11.23 (3.85, 29.49)	37.5%	1.40 (0.10, 4.95)	62.5%	0.03 (0.01, 0.20)	0.0%	0.01 (0.01, 0.11)

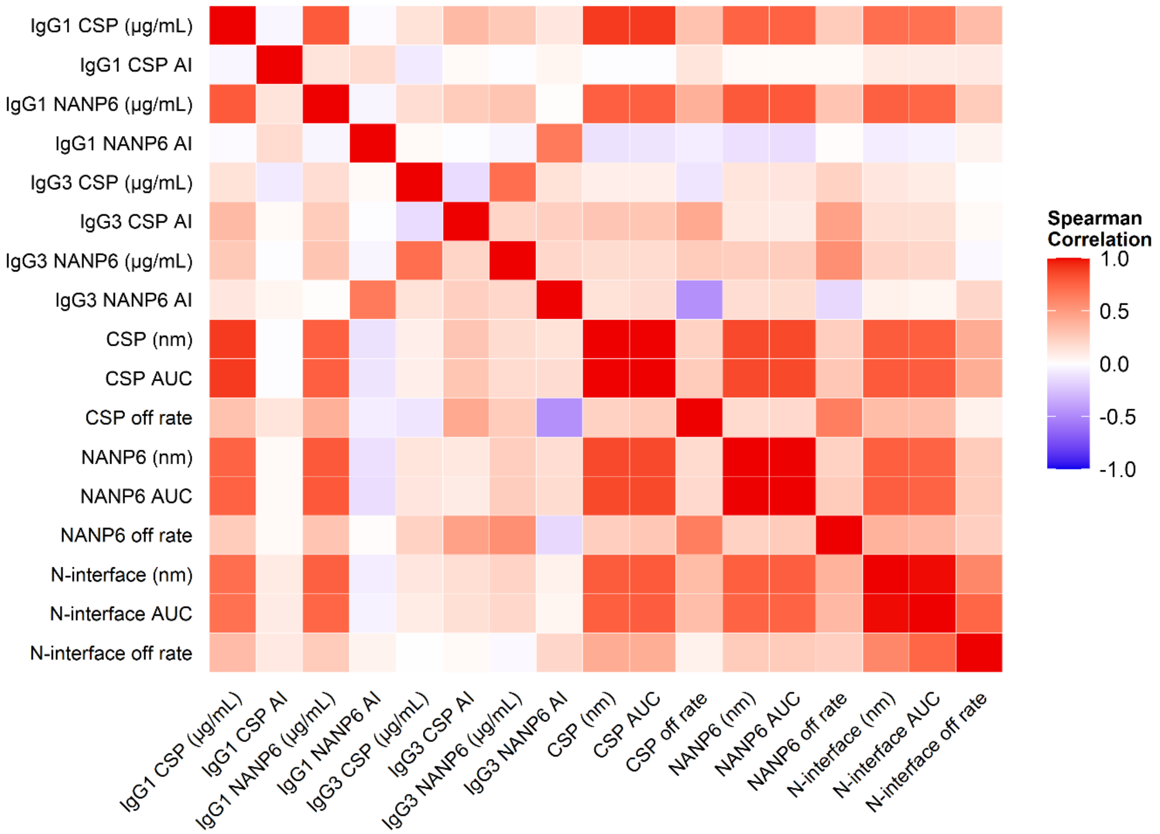

Supplemental Figure 6. Correlation of humoral immune measures. A heatmap of Spearman's rank correlation coefficients between measurements across the MAL092 and MAL102 studies is displayed. Intra-assay correlations are shown in the upper left quadrant (BAMA IgG subclass measurements) and lower right quadrant (BLI serum measurements). Inter-assay correlations are shown in the upper right and lower left quadrants.

Supplemental Table 4. Correlation of humoral immune measures. Spearman's rank correlation coefficients between measurements across the MAL092 and MAL102 studies as displayed in S6 Fig. ns = non-significant (P>0.05).

	IgG1 CSP (µg/mL)	IgG1 CSP AI	IgG1 NANP6 (µg/mL)	IgG1 NANP6 AI	IgG3 CSP (µg/mL)	IgG3 CSP AI	IgG3 NANP6 (µg/mL)	IgG3 NANP6 AI	CSP (nm)	CSP AUC	CSP off rate	NANP6 (nm)	NANP6 AUC	NANP6 off rate	N-interface (nm)	N-interface AUC	N-interface off rate
IgG1 CSP (µg/mL)	r=1.00 (P<0.001)	ns	r=0.79 (P<0.001)	ns	ns	r=0.35 (P=0.023)	r=0.27 (P=0.007)	ns	r=0.90 (P<0.001)	r=0.90 (P<0.001)	r=0.31 (P=0.012)	r=0.76 (P<0.001)	r=0.76 (P<0.001)	ns	r=0.71 (P<0.001)	r=0.69 (P<0.001)	r=0.34 (P=0.036)
IgG1 CSP AI	ns	r=1.00 (P<0.001)	ns	ns	ns	ns	ns	ns	ns	ns	ns	ns	ns	ns	ns	ns	ns
IgG1 NANP6 (µg/mL)	r=0.79 (P<0.001)	ns	r=1.00 (P<0.001)	ns	ns	ns	r=0.29 (P=0.004)	ns	r=0.77 (P<0.001)	r=0.77 (P<0.001)	r=0.40 (P=0.001)	r=0.80 (P<0.001)	r=0.80 (P<0.001)	ns	r=0.77 (P<0.001)	r=0.74 (P<0.001)	ns
IgG1 NANP6 AI	ns	ns	ns	r=1.00 (P<0.001)	ns	ns	ns	r=0.66 (P=0.008)	ns	ns	ns	ns	ns	ns	ns	ns	ns
IgG3 CSP (µg/mL)	ns	ns	ns	ns	r=1.00 (P<0.001)	ns	r=0.70 (P<0.001)	ns	ns	ns	ns	ns	ns	ns	ns	ns	ns
IgG3 CSP AI	r=0.35 (P=0.023)	ns	ns	ns	ns	r=1.00 (P<0.001)	ns	ns	ns	ns	r=0.43 (P=0.017)	ns	ns	r=0.47 (P=0.031)	ns	ns	ns
IgG3 NANP6 (µg/mL)	r=0.27 (P=0.007)	ns	r=0.29 (P=0.004)	ns	r=0.70 (P<0.001)	ns	r=1.00 (P<0.001)	ns	ns	ns	r=0.26 (P=0.039)	r=0.25 (P=0.015)	r=0.25 (P=0.014)	r=0.56 (P<0.001)	r=0.22 (P=0.037)	ns	ns
IgG3 NANP6 AI	ns	ns	ns	r=0.66 (P=0.008)	ns	ns	ns	r=1.00 (P<0.001)	ns	ns	ns	ns	ns	ns	ns	ns	ns
CSP (nm)	r=0.90 (P<0.001)	ns	r=0.77 (P<0.001)	ns	ns	ns	ns	ns	r=1.00 (P<0.001)	r=1.00 (P<0.001)	ns	r=0.85 (P<0.001)	r=0.86 (P<0.001)	ns	r=0.79 (P<0.001)	r=0.77 (P<0.001)	r=0.42 (P=0.010)
CSP AUC	r=0.90 (P<0.001)	ns	r=0.77 (P<0.001)	ns	ns	ns	ns	ns	r=1.00 (P<0.001)	r=1.00 (P<0.001)	r=0.26 (P=0.035)	r=0.85 (P<0.001)	r=0.86 (P<0.001)	ns	r=0.79 (P<0.001)	r=0.78 (P<0.001)	r=0.41 (P=0.011)
CSP off rate	r=0.31 (P=0.012)	ns	r=0.40 (P=0.001)	ns	ns	r=0.43 (P=0.017)	r=0.26 (P=0.039)	ns	ns	r=0.26 (P=0.035)	r=1.00 (P<0.001)	ns	ns	r=0.64 (P<0.001)	r=0.34 (P=0.006)	r=0.33 (P=0.008)	ns
NANP6 (nm)	r=0.76 (P<0.001)	ns	r=0.80 (P<0.001)	ns	ns	ns	r=0.25 (P=0.015)	ns	r=0.85 (P<0.001)	r=0.85 (P<0.001)	ns	r=1.00 (P<0.001)	r=1.00 (P<0.001)	ns	r=0.77 (P<0.001)	r=0.75 (P<0.001)	ns
NANP6 AUC	r=0.76 (P<0.001)	ns	r=0.80 (P<0.001)	ns	ns	ns	r=0.25 (P=0.014)	ns	r=0.86 (P<0.001)	r=0.86 (P<0.001)	ns	r=1.00 (P<0.001)	r=1.00 (P<0.001)	ns	r=0.77 (P<0.001)	r=0.75 (P<0.001)	ns
NANP6 off rate	ns	ns	ns	ns	ns	r=0.47 (P=0.031)	r=0.56 (P<0.001)	ns	ns	ns	r=0.64 (P<0.001)	ns	ns	r=1.00 (P<0.001)	r=0.38 (P=0.012)	r=0.36 (P=0.020)	ns
N-interface (nm)	r=0.71 (P<0.001)	ns	r=0.77 (P<0.001)	ns	ns	ns	r=0.22 (P=0.037)	ns	r=0.79 (P<0.001)	r=0.79 (P<0.001)	r=0.34 (P=0.006)	r=0.77 (P<0.001)	r=0.77 (P<0.001)	r=0.38 (P=0.012)	r=1.00 (P<0.001)	r=0.99 (P<0.001)	r=0.60 (P<0.001)
N-interface AUC	r=0.69 (P<0.001)	ns	r=0.74 (P<0.001)	ns	ns	ns	ns	ns	r=0.77 (P<0.001)	r=0.78 (P<0.001)	r=0.33 (P=0.008)	r=0.75 (P<0.001)	r=0.75 (P<0.001)	r=0.36 (P=0.020)	r=0.99 (P<0.001)	r=1.00 (P<0.001)	r=0.74 (P<0.001)
N-interface off rate	r=0.34 (P=0.036)	ns	ns	ns	ns	ns	ns	ns	r=0.42 (P=0.010)	r=0.41 (P=0.011)	ns	ns	ns	ns	r=0.60 (P<0.001)	r=0.74 (P<0.001)	r=1.00 (P<0.001)

Supplemental Table 5. Additional univariate logistic regression results. Odds ratios, 95% confidence intervals, and raw and FDR-adjusted P-values obtained from unadjusted logistic regression models (Protection ~ immune measurement) and demographics and regimen-adjusted logistic regression models (Protection ~ Regimen + Age (years) + Sex + immune measurement) fit independently to each immune measurement based on MAL092 day of CHMI data. Age and sex terms did not contribute significantly to any model ($p > 0.05$). Associations were considered statistically significant for FDR-adjusted P-values < 0.2 (measurements in bold).

Model Adjustment	Measurement	Odds Ratio (95% CI)	P-value	FDR-adjusted P-value
Regimen, age, sex	**IgG1 CSP 334-hIgG1 equivalent**	2.75 (1.61, 5.07)	0.0005	0.0020
	**IgG1 NANP6 334-hIgG1 equivalent**	2.63 (1.54, 4.90)	0.0009	0.0027
	IgG3 CSP 334-hIgG3 equivalent	1.35 (0.87, 2.12)	0.1831	0.2746
	IgG3 NANP6 334-hIgG3 equivalent	0.95 (0.61, 1.49)	0.8213	0.8314
	IgG1 CSP AI	1.06 (0.68, 1.67)	0.7844	0.8314
	IgG1 NANP6 AI	1.06 (0.58, 1.87)	0.8314	0.8314
	IgG3 CSP AI	1.89 (0.83, 4.99)	0.1563	0.2679
	IgG3 NANP6 AI	1.21 (0.29, 5.07)	0.7809	0.8314
	**Serum CSP AUC_{diss}**	2.41 (1.46, 4.29)	0.0012	0.0029
	**Serum NANP6 AUC_{diss}**	3.21 (1.74, 6.57)	0.0005	0.0020
	**Serum N Interface AUC_{diss}**	2.62 (1.61, 4.50)	0.0002	0.0020
Unadjusted	**IgG1 CSP 334-hIgG1 equivalent**	2.12 (1.35, 3.50)	0.0017	0.0042
	**IgG1 NANP6 334-hIgG1 equivalent**	2.59 (1.58, 4.65)	0.0005	0.0017
	IgG3 CSP 334-hIgG3 equivalent	1.32 (0.87, 2.02)	0.1937	0.3551
	IgG3 NANP6 334-hIgG3 equivalent	1.05 (0.70, 1.61)	0.8106	0.8916
	IgG1 CSP AI	1.08 (0.71, 1.66)	0.7013	0.8916
	IgG1 NANP6 AI	1.03 (0.59, 1.75)	0.9150	0.9150
	IgG3 CSP AI	1.38 (0.74, 2.78)	0.3167	0.4977
	IgG3 NANP6 AI	1.19 (0.38, 3.48)	0.7422	0.8916
	**Serum CSP AUC_{diss}**	2.18 (1.37, 3.70)	0.0019	0.0042
	**Serum NANP6 AUC_{diss}**	3.22 (1.87, 6.13)	0.0001	0.0006
	**Serum N Interface AUC_{diss}**	2.71 (1.70, 4.51)	0.0001	0.0006

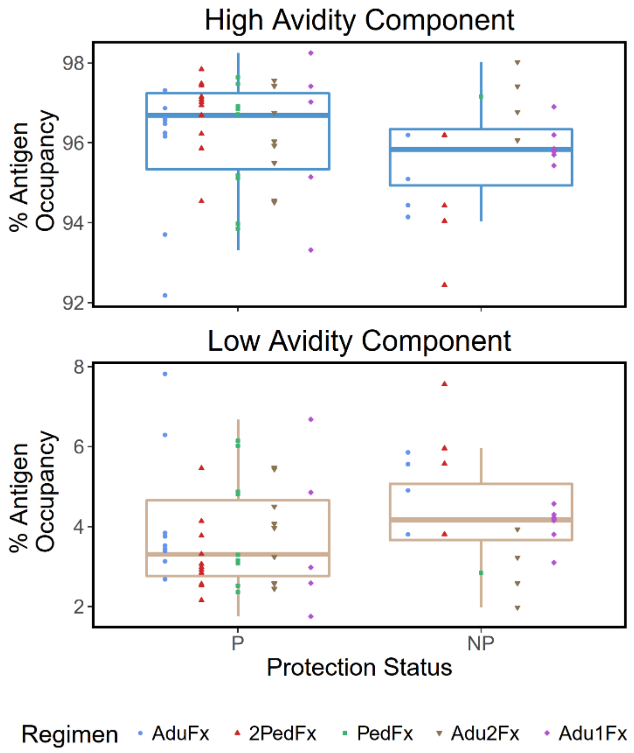

Supplemental Figure 7. Protected RTS,S/AS01 vaccinees have higher antigen occupancy by high avidity antibodies. The antigen occupancy by RTS,S-induced high avidity antibodies (top row) and low avidity antibodies (bottom row) are shown for PfCSP binding for MAL092 vaccinees' sera drawn at day 286 (90 days post-final immunization or day of 1st CHMI). The heterogeneity in avidity of vaccinees' serum antibodies were resolved using PAART. The difference in antigen occupancy of the high avidity component was not significantly different between protected and non-protected vaccinees ($p = 0.073$, Mann-Whitney *U* test).

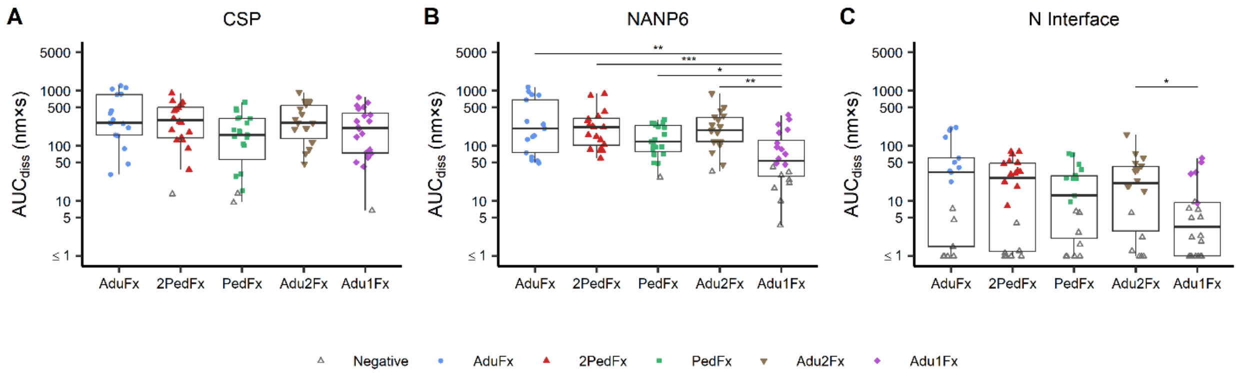

Supplemental Figure 8. Serum Ig magnitude and avidity by regimen. The AUC_{diss} values of vaccinees' MAL092 DoC (day 286, which is 90 days post-final immunization or day of 1st CHMI) serum interaction with CSP (**A**), NANP6 (**B**), and N Interface (**C**) are shown. Group comparisons with significant differences are indicated (* $P < 0.05$, ** $P < 0.01$, *** $P < 0.001$ by two-sided Mann-Whitney *U* test). All pairwise group comparisons were performed for all panels, and unless otherwise indicated, $p > 0.05$.

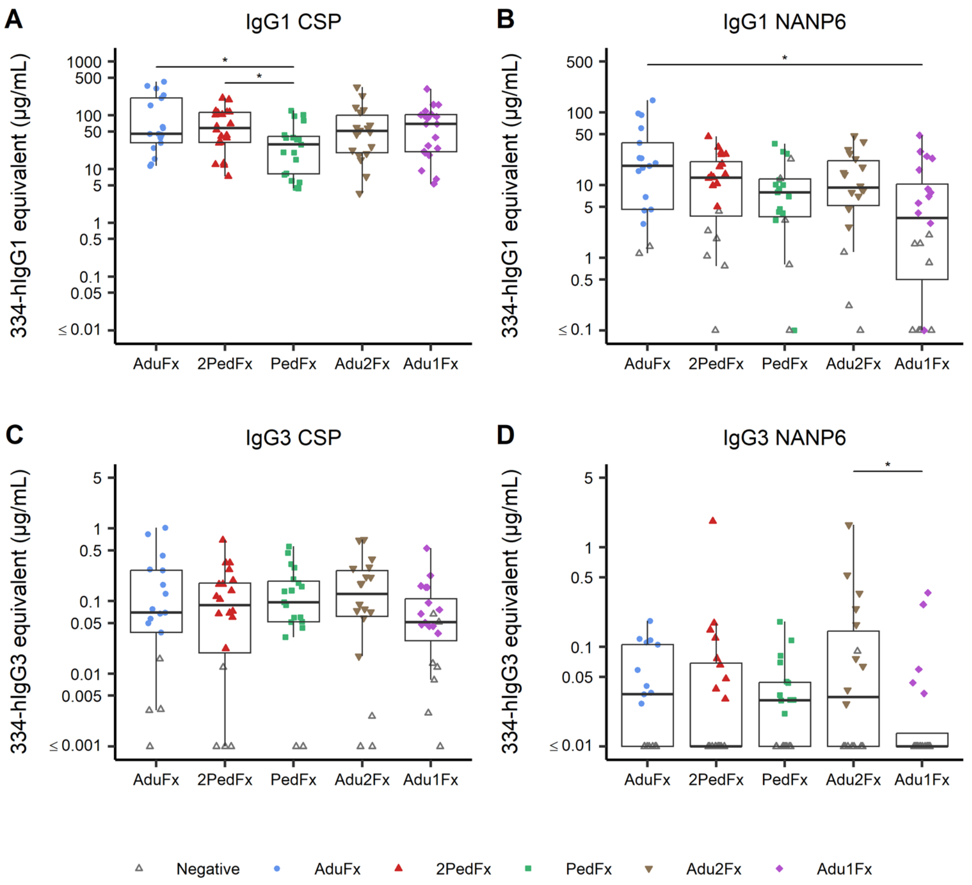

Supplemental Figure 9. Antibody subclass binding magnitude by regimen. The 334-hIgG1 or hIgG3 equivalent concentrations in vaccinee sera from MAL092 DoC (day 286) as in S8 Fig for IgG1 CSP (**A**), IgG1 NANP6 (**B**), IgG3 CSP (**C**), and IgG3 NANP6 (**D**) are shown. Group comparisons with significant differences are indicated (* $P < 0.05$ by two-sided Mann-Whitney *U* test). All pairwise group comparisons were performed for all panels, and unless otherwise indicated, $p > 0.05$. Note that all panels have different y-axis scales to best show group differences.

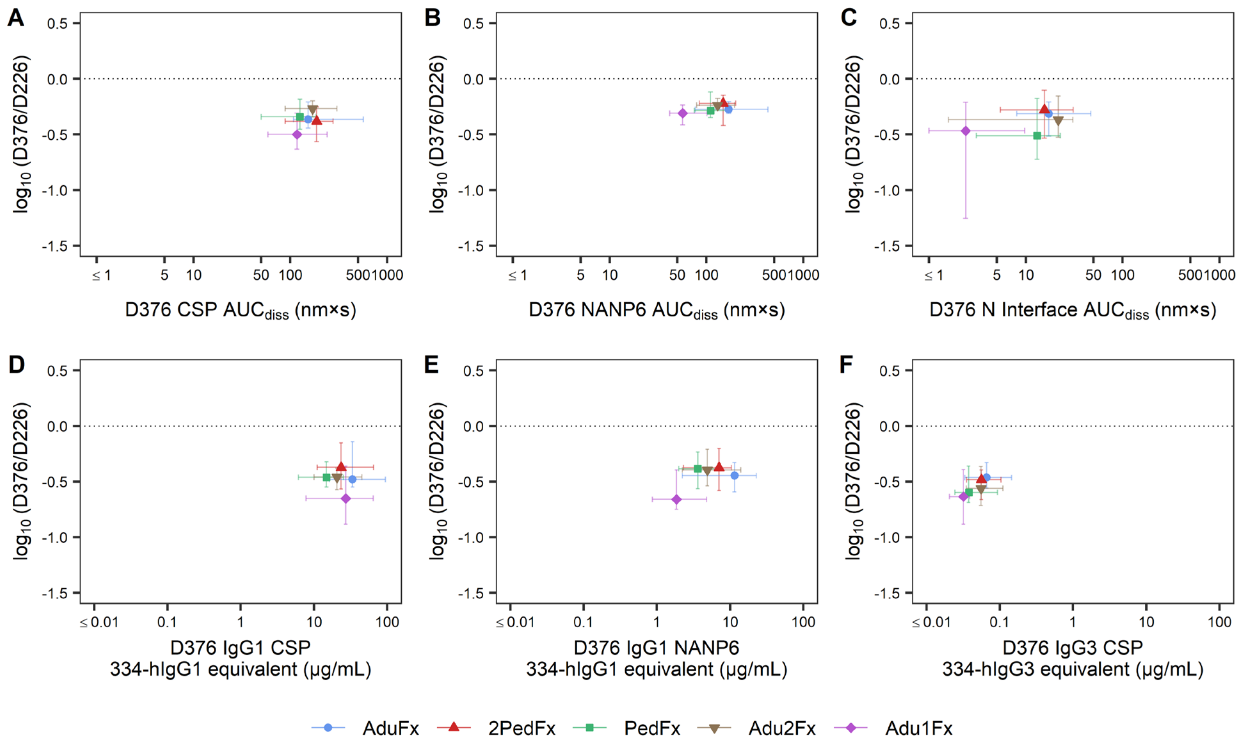

Supplemental Figure 10. Six month durability of RTS,S/AS01 induced antibody responses. A plot of fold change in antibody responses in MAL092 from day 226 (30 days post-final immunization) to day 376 (180 days post-final immunization or 90 days post-1st CHMI) vs. day 376 antibody responses by RTS,S/AS01 regimen is shown for PfCSP-specific (**A**), NANP6-specific (**B**), and N Interface-specific (**C**) AUC_{diss}, IgG1 PfCSP (**D**), IgG1 NANP6 (**E**), and IgG3 CSP (**F**) 334-hIgG1/hIgG3 equivalent concentration. For each regimen, individual points represent median and error bars represent 25th to 75th percentiles. Horizontal line represents no change in antibody response from day 226 to day 376.
